# Prevalence and outcomes of atrial fibrillation in hospitalizations during pregnancy and puerperium

**DOI:** 10.3389/fcvm.2026.1814450

**Published:** 2026-07-30

**Authors:** Zhenyu Zeng, Suxuan Liu, Shengyong Wu, Feifei Dong, Fei Luo, Li Quan, Chenxin Chen, Xudong Xu, Zhifu Guo, Cheng Wu

**Affiliations:** 1 Department of Cardiology, The First Affiliated Hospital (Changhai Hospital) of Naval Medical University, Shanghai, China; 2 Department of Military Health Statistics, Naval Medical University, Shanghai, China; 3 Department of Cardiology, Shanghai Eastern Hepatobiliary Surgery Hospital, Naval Medical University, Shanghai, China

**Keywords:** atrial fibrillation, CHA_2_DS_2_-VASc, pregnancy, prevalence, puerperium

## Abstract

**Background:**

Atrial fibrillation (AF) is a common cardiac arrhythmia, but data on the impact during delivery remains limited.

**Objectives:**

The aim of the study is to examine a) the prevalence of AF during pregnancy and puerperium, b) the association between AF and adverse outcomes, and c) the association between adverse cardiovascular outcomes and CHA_2_DS_2_-VASc scores in patients with AF.

**Methods:**

The National Inpatient Sample (NIS) database was used to identify pregnancy-related hospitalizations between 2008 and 2017. Adverse outcomes included: in-hospital mortality, stroke, acute heart failure, cardiogenic shock, acute kidney failure, and acute myocardial infarction. Logistic models were constructed to explore the relationship between AF and adverse outcomes.

**Results:**

Of 40,996,860 pregnancy- and puerperium-related hospitalizations, 15,162 (0.037%) also had AF. The prevalence of AF increased from 2.18 (95%CI = 2.04–2.31) per 10,000 hospitalizations in 2008 to 4.87 (95%CI = 4.65–5.09) per 10,000 in 2017 (*P*_trend_ < 0.001). The proportion of patients with AF increased with advanced maternal age (*P*_trend_ < 0.001). The prevalence of AF was higher in Black individuals, those from low-income households, and those with advanced maternal age (*P*_trend_ < 0.001). Maternal patients with AF had significantly higher in-hospital mortality (OR = 2.02, 95%CI = 1.56–2.61, *P* < 0.001). Stroke, acute myocardial infarction, acute heart failure, and cardiogenic shock were associated with increasing CHA_2_DS_2_-VASc scores in patients with AF (*P*_trend_ < 0.001).

**Conclusions:**

The prevalence of AF has been increasing in the pregnant and puerperium population and is associated with adverse outcomes. Higher CHA_2_DS_2_-VASc scores are associated with adverse outcomes in maternal patients with AF.

## Introduction

Atrial fibrillation (AF) is the most common tachyarrhythmia and significantly increases the risk of severe cardiovascular complications, such as stroke, myocardial infarction, and heart failure. AF is becoming a public health problem with a substantial financial burden (1). Pregnancy is associated with physiological changes in the cardiovascular system, including structural and hemodynamic alterations, which might increase the risk of women suffering from AF. AF during pregnancy and puerperium can result in a high risk of embolic events and hemodynamic instability, and is devastating for maternal and fetal outcomes. Nevertheless, there are limited studies investigating the prevalence and outcomes in patients with AF during pregnancy and puerperium.

The CHA_2_DS_2_-VASc [C: congestive heart failure, H: hypertension, A: age of ≥75 years (doubled), D: diabetes mellitus, S: previous stroke (doubled), V: vascular disease, A: age between 65 and 74 years, Sc: female gender] scoring system is used in non-valvular AF patients to stratify the risk of thromboembolism and guide the anticoagulation strategy (2). However, maternal patients were excluded from the CHA_2_DS_2_-VASc application. Limited research has been conducted on using the value of the CHA_2_DS_2_-VASc score in maternal patients.

We aimed to fill the knowledge gaps by investigating the prevalence of AF during pregnancy and puerperium and its association with adverse outcomes. We also assessed the association between CHA_2_DS_2_-VASc scores and adverse outcomes in maternal patients with AF.

## Methods

### Data source

The National Inpatient Sample (NIS) data sets, which are publicly available and cover approximates a 20% stratified sample of discharges from U.S. community hospitals, were used to conduct this study. This database is sponsored by the Agency for Healthcare and Research and Quality and encompasses the brief diagnosis and treatment information abstracted from discharges from 47 U.S. states, covering >97% of the U.S. population (3). Patient demographic characteristics were collected from the State Inpatient Databases (SID) of the United States, which were currently contributed to Healthcare Cost and Utilization Project (HCUP) (4). According to the HCUP guidelines, this study does not require ethical approval because all data from the NIS were de-identified (3).

### Study population

**Figure 1:**
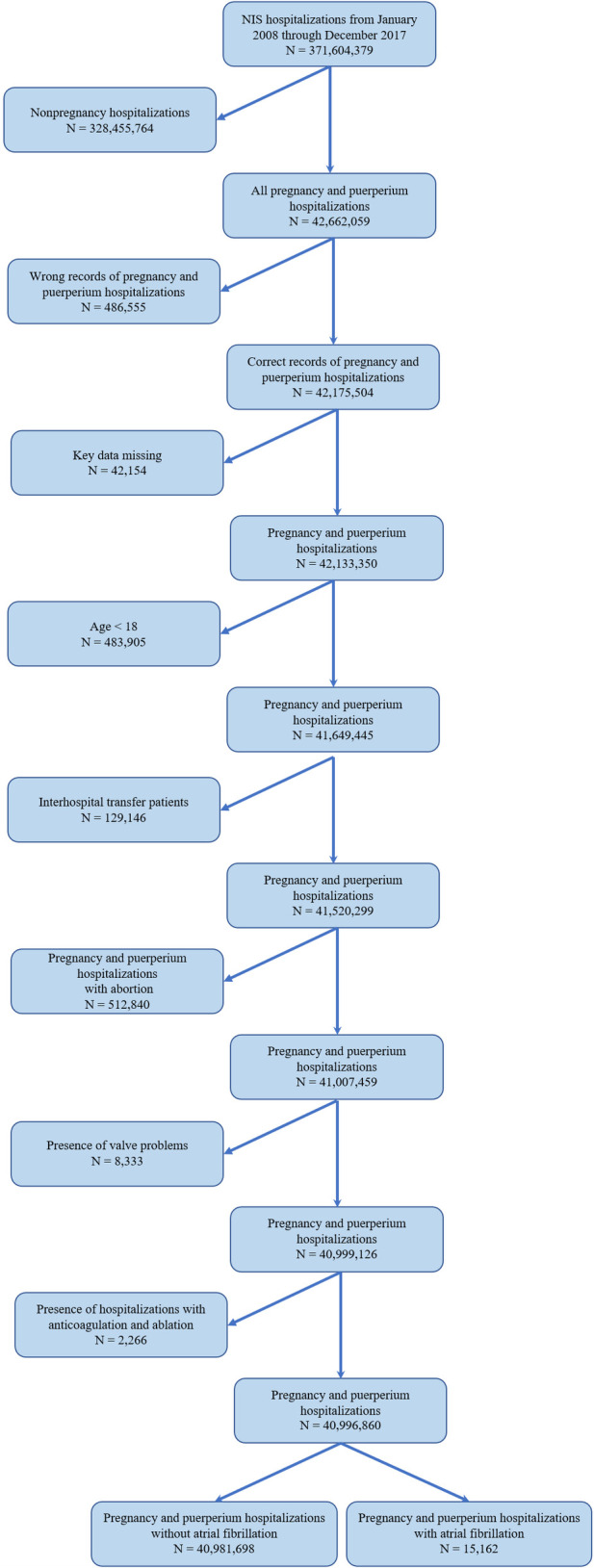
Selection flow ydiagram of target population. Selection flow diagram of target population. NIS, National Inpatient Sample.

The NIS database was queried for all patients during pregnancy and puerperium (6 weeks after delivery) from January 1, 2008, to December 31, 2017. Of 371,604,379 patients in the NIS between 2008 and 2017, all hospitalizations aged ≥18 years who had diagnoses related to pregnancy, labor, or postpartum period were identified using the International Classification of Diseases-9th Revision-Clinical Modification (ICD-9-CM) diagnostic or ICD-10-CM diagnostic. AF was defined as the diagnosis of patients, including the code of atrial fibrillation, using ICD-9-CM or ICD-10-CM (Supplement Table S1). The selection steps are shown in Figure 1.

### Outcomes measures

The primary outcomes included: 1) the temporal trends in the prevalence of AF during pregnancy and puerperium; 2) the difference in adverse outcomes between hospitalizations with and without AF.

The secondary outcomes included: 1) risk factors associated with AF during pregnancy and puerperium; 2) the association of AF with adverse outcomes, cost, and length of stay (LOS); 3) the relationship between the stratification of CHA_2_DS_2_-VASc and adverse cardiovascular events of hospitalizations during pregnancy and puerperium with AF.

### Statistical analysis

Continuous variables were presented as mean (standard deviation, SD) or median (interquartile range, IQR) depending on their distribution. Categorical variables were represented as the number *n* (%). The characteristics and demographics were compared between patients with and without AF. Using weights provided by NIS, we calculated the overall prevalence of AF during pregnancy and puerperium. The prevalence was examined in subgroups, including race, household income, hospital region and age. The Cochran-Armitage trend test was used to evaluate the significance of trends over time and the CHA_2_DS_2_-VASc score. Univariate analysis of the comparisons between two groups was determined by Student's *t*-test or Mann–Whitney *U*-test for normal distributed or non-normal distributed continuous variables, and Pearson Chi-Square test or Mann–Whitney *U*-test for unordered categorical variable or ordinal categorical variable.

Using the covariates, logistic models were constructed to explore the relationship between AF and adverse outcomes. We employed a multi-model analytical strategy: Models 1 and 2 utilized stepwise logistic regression (entry *p* < 0.05, removal *p* < 0.10). Model 1 incorporated the individual clinical components of CHA_2_DS_2_-VASc score as composite covariates, whereas Model 2 integrated the CHA_2_DS_2_-VASc score as a composite covariate. Model 3 addressed missing data through multiple imputation via chained equations (MICE, 5 datasets) with Rubin's rule pooling, while Model 4 applied propensity score matching (1:1 nearest-neighbor, caliper = 0.2*σ*), covariate balance before and after matching was assessed using standardized mean differences, with a value <0.1 indicating acceptable balance(Supplement Table S10). Model 1 used the data after missing variables were imputed, while model 2 used the original data. To evaluate whether the diseases related to the cost and LOS of the hospitalization, we constructed multivariate linear models, adjusting for the same covariate assessment as model 1. Univariate and two multivariate ogistic models were constructed to explore the clinical factors associated with AF as model 1 and model 2 in all cohort and high-prevalence subgroups. To examine heterogeneity in the predictive accuracy of the CHA₂DS₂-VASc score, we calculated the area under the receiver operating characteristic curve (AUC) and its 95% confidence interval for the outcome of in-hospital mortality within strata of age, race and ethnicity. All tests were two-tailed, and *P* < 0.05 was considered significant unless otherwise specified. Statistical analysis was performed using SAS version 9.4 (SAS Institute Inc).

### Missing variables

Most values were missing for <2% of all hospitalizations, while race and cost were missing for 9.36% and 2.17%. The missing data of the categorical variables were imputed by the dominant category and the continuous variables by the median (5, 6).

## Results

Among 40,996,860 hospitalizations for pregnancy and puerperium from 2008 to 2017, AF was diagnosed in 15,162 patients (0.037%) (Figure 1). After direct age standardization to the 2024 U.S. female population, the age-adjusted incidence was 0.038%. A comparison of demographics and hospital characteristics between maternal patients with and without AF was presented (Table 1). Compared to patients without AF, maternal patients with AF had a higher likelihood of being Black individuals, maternal age over 35 years, and having the lowest median household income quartile, as well as presenting with tobacco abuse, obstructive sleep apnea, lipid metabolism disorder, obesity, hypertension, diabetes, and a higher CHA_2_DS_2_-VASc score (all *P* < 0.001).

**Table 1:** Comparison of demographics, hospital characteristics, interventions between hospitalization with and without atrial fibrillation.

Variables	All cohort	Atrial fibrillation		*P* value
	*N* = 40,996,860	Absent	Present	
		*N* = 40,981,698	*N* = 15,162	
Age in years at admission	28.32 ± 12.95	28.32 ± 12.94	31.16 ± 14.96	<.001
Age group				
18∼24	11,922,245 (29.08)	11,919,623 (29.09)	2,622 (17.29)	<.001
25∼29	11,936,750 (29.12)	11,932,895 (29.12)	3,855 (25.43)	
30∼34	10,693,025 (26.08)	10,688,930 (26.08)	4,094 (27.00)	
35∼39	5,208,208 (12.70)	5,205,235 (12.70)	2,973 (19.61)	
40∼44	1,154,771 (2.82)	1,153,642 (2.82)	1,129 (7.45)	
≥45	81,861 (0.20)	81,372 (0.20)	489 (3.22)	
Race				
White	23,412,509 (57.11)	23,404,557 (57.11)	7,952 (52.45)	<.001
Black	5,595,022 (13.65)	5,590,464 (13.64)	4,558 (30.06)	
Hispanic	7,869,837 (19.20)	7,868,238 (19.20)	1,598 (10.54)	
Other	4,119,493 (10.05)	4,118,439 (10.05)	1,054 (6.95)	
Elderly parturient women^a^	6,444,840 (15.72)	6,440,250 (15.71)	4,590 (30.28)	<.001
Admission day is on a weekend	8,005,528 (19.53)	8,002,577 (19.53)	2,951 (19.47)	0.8497
Elective admission	19,590,492 (47.79)	19,58,5,489 (47.79)	5,002 (32.99)	<.001
Season				
Winter	9,586,369 (23.38)	9,582,773 (23.38)	3,596 (23.72)	0.1568
Spring	9,820,061 (23.95)	9,816,344 (23.95)	3,717 (24.51)	
Summer	11,57,1,708 (28.23)	11,567,520 (28.23)	4,187 (27.62)	
Autumn	10,018,722 (24.44)	10,015,061 (24.44)	3,661 (24.15)	
Control/ownership of hospital				
Government nonfederal	5,252,299 (12.81)	5,250,294 (12.81)	2,005 (13.22)	<.001
Private not for profit	30,049,144 (73.30)	30,037,648 (73.30)	11,496 (75.82)	
Private investor owned	5,695,418 (13.89)	5,693,757 (13.89)	1,660 (10.95)	
Bed size of hospital				
Small	5,414,371 (13.21)	5,413,038 (13.21)	1,333 (8.79)	<.001
Medium	11,593,007 (28.28)	11,589,461 (28.28)	3,546 (23.38)	
Large	23,989,482 (58.52)	23,979,199 (58.51)	10,283 (67.82)	
Teaching status of hospital				
Nonteaching	18,086,372 (44.12)	18,081,975 (44.12)	4,397 (29.00)	<.001
Teaching	22,910,488 (55.88)	22,899,723 (55.88)	10,765 (71.00)	
Location status of hospital				
Rural	4,199,874 (10.24)	4,199,103 (10.25)	771 (5.08)	<.001
Urban	36,796,986 (89.76)	36,782,595 (89.75)	14,391 (94.92)	
Region of hospital				
Northeast	6,659,219 (16.24)	6,656,547 (16.24)	2,672 (17.62)	<.001
Midwest	8,702,310 (21.23)	8,699,055 (21.23)	3,255 (21.47)	
South	15,716,954 (38.34)	15,710,679 (38.34)	6,275 (41.39)	
West	9,918,377 (24.19)	9,915,418 (24.19)	2,960 (19.52)	
Primary expected payer				
Medicare	326,638 (0.80)	325,793 (0.79)	845 (5.57)	<.001
Medicaid	17,702,974 (43.18)	17,696,619 (43.18)	6,355 (41.91)	
Private insurance	20,555,598 (50.14)	20,548,425 (50.14)	7,172 (47.31)	
Self-pay	11,80,545 (2.88)	1,180,141 (2.88)	404 (2.66)	
Other	1,231,105 (3.00)	1,230,720 (3.00)	386 (2.55)	
Median household income percentile				
<P25	11,900,816 (29.03)	11,895,682 (29.03)	5,134 (33.86)	<.001
P25∼P49	10,155,987 (24.77)	10,152,453 (24.77)	3,534 (23.31)	
P50∼P74	9,982,573 (24.35)	9,979,269 (24.35)	3,304 (21.79)	
≥P75	8,957,484 (21.85)	8,954,294 (21.85)	3,190 (21.04)	
Tobacco abuse	33,25,478 (8.11)	3,323,154 (8.11)	2,324 (15.33)	<.001
Alcohol abuse	67,054 (0.16)	66,978 (0.16)	75 (0.50)	<.001
Drug abuse	834,081 (2.03)	833,458 (2.03)	623 (4.11)	<.001
Obstructive sleep apnea	30,988 (0.08)	30,594 (0.07)	393 (2.59)	<.001
Disorders of lipoid metabolism	81,150 (0.20)	80,603 (0.20)	547 (3.61)	<.001
Nephrotic syndrome	4,922 (0.01)	4,918 (0.01)	5 (0.03)	0.0333
Chronic kidney disease	36,433 (0.09)	36,024 (0.09)	409 (2.70)	<.001
Thyrotoxicosis	76,189 (0.19)	75,869 (0.19)	320 (2.11)	<.001
Varicose vein of lower limb	60,487 (0.15)	60,473 (0.15)	14 (0.09)	0.0887
Acquired immune deficiency syndrome	10,873 (0.03)	10,858 (0.03)	15 (0.10)	<.001
Deficiency anemias	3,469,643 (8.46)	3,467,270 (8.46)	2,373 (15.65)	<.001
Rheumatoid arthritis/collagen vascular diseases	125,060 (0.31)	124,842 (0.30)	218 (1.44)	<.001
Chronic blood loss anemia	4,735,410 (11.55)	4,732,536 (11.55)	2,874 (18.95)	<.001
Chronic pulmonary disease	1,714,902 (4.18)	1,713,147 (4.18)	1,755 (11.58)	<.001
Coagulopathy	733,422 (1.79)	732,617 (1.79)	805 (5.31)	<.001
Depression	1,015,765 (2.48)	1,014,671 (2.48)	1,094 (7.21)	<.001
Hypertension	1,016,069 (2.48)	1,013,518 (2.47)	2,551 (16.83)	<.001
Hypothyroidism	1,111,936 (2.71)	1,110,896 (2.71)	1,040 (6.86)	<.001
Liver disease	89,548 (0.22)	89,448 (0.22)	100 (0.66)	<.001
Lymphoma	6,817 (0.02)	6,812 (0.02)	5 (0.03)	0.1184
Fluid and electrolyte disorders	495,529 (1.21)	493,545 (1.20)	1,984 (13.09)	<.001
Metastatic cancer	3,061 (0.01)	3,061 (0.01)	0 (0.00)	0.2872
Other neurological disorders	357,436 (0.87)	356,917 (0.87)	519 (3.42)	<.001
Obesity	2,568,669 (6.27)	2,565,968 (6.26)	2,701 (17.82)	<.001
Paralysis	21,771 (0.05)	21,717 (0.05)	54 (0.36)	<.001
Peripheral vascular disorders	8,407 (0.02)	8,326 (0.02)	81 (0.53)	<.001
Psychoses	435,042 (1.06)	434,639 (1.06)	403 (2.65)	<.001
Pulmonary circulation disorders	19,720 (0.05)	19,415 (0.05)	305 (2.01)	<.001
Solid tumor without metastasis	11,263 (0.03)	11,244 (0.03)	19 (0.13)	<.001
Peptic ulcer disease excluding bleeding	2,055 (0.01)	2,050 (0.01)	5 (0.03)	<.001
Valvular disease	119,957 (0.29)	118,903 (0.29)	1,054 (6.95)	<.001
Weight loss	36,860 (0.09)	36,658 (0.09)	202 (1.33)	<.001
Pre-eclampsia/eclampsia	1,860,449 (4.54)	1,859,614 (4.54)	835 (5.50)	<.001
CHA_2_DS_2_-VASc				<.001
Hypertension	4,413,999 (10.77)	4,410,133 (10.76)	3,867 (25.50)	<.001
Diabetes	1,207,027 (2.94)	1,205,993 (2.94)	1,033 (6.82)	<.001
Peripheral Vascular Disease	19,725 (0.05)	19,395 (0.05)	330 (2.18)	<.001
CHF	52,918 (0.13)	51,136 (0.12)	1,782 (11.75)	<.001
Stroke/TIA	37,475 (0.09)	37,067 (0.09)	408 (2.69)	<.001
CHA_2_DS_2_-VASc Score	1.00 (1.00–1.00)	1.00 (1.00–1.00)	1.00 (1.00–2.00)	<.001
Length of stay days	2.00 (2.00–3.00)	2.00 (2.00–3.00)	3.00 (2.00–4.00)	<.001
Patient disposition				<.001
Routine	40,048,372 (97.69)	40,034,546 (97.69)	13,826 (91.19)	
Transfer to Short-term Hospital	42,178 (0.10)	42,101 (0.10)	77 (0.51)	
Transfer to skilled nursing facility or intermediate care facility	43,780 (0.11)	43,596 (0.11)	184 (1.21)	
Home Health Care	741,324(1.81)	740,568(1.81)	757(4.99)	
Against Medical Advice	116,284(0.28)	116,073(0.28)	210(1.39)	
In-hospital death	4,274(0.01)	4,167(0.01)	108(0.71)	
Discharge alive destination unknown	647(0.00)	647(0.00)	0(0.00)	

a Elderly parturient women were defined as female during pregnancy or puerperium whose age older than 35-year-old.

Causing the weight of observation may not be integer, the count of each group may less or more than the total of all group after rounding off. CHF,Chronic heart failure;TIA,Transient ischemic attack.

### Primary outcomes

**Figure 2:**
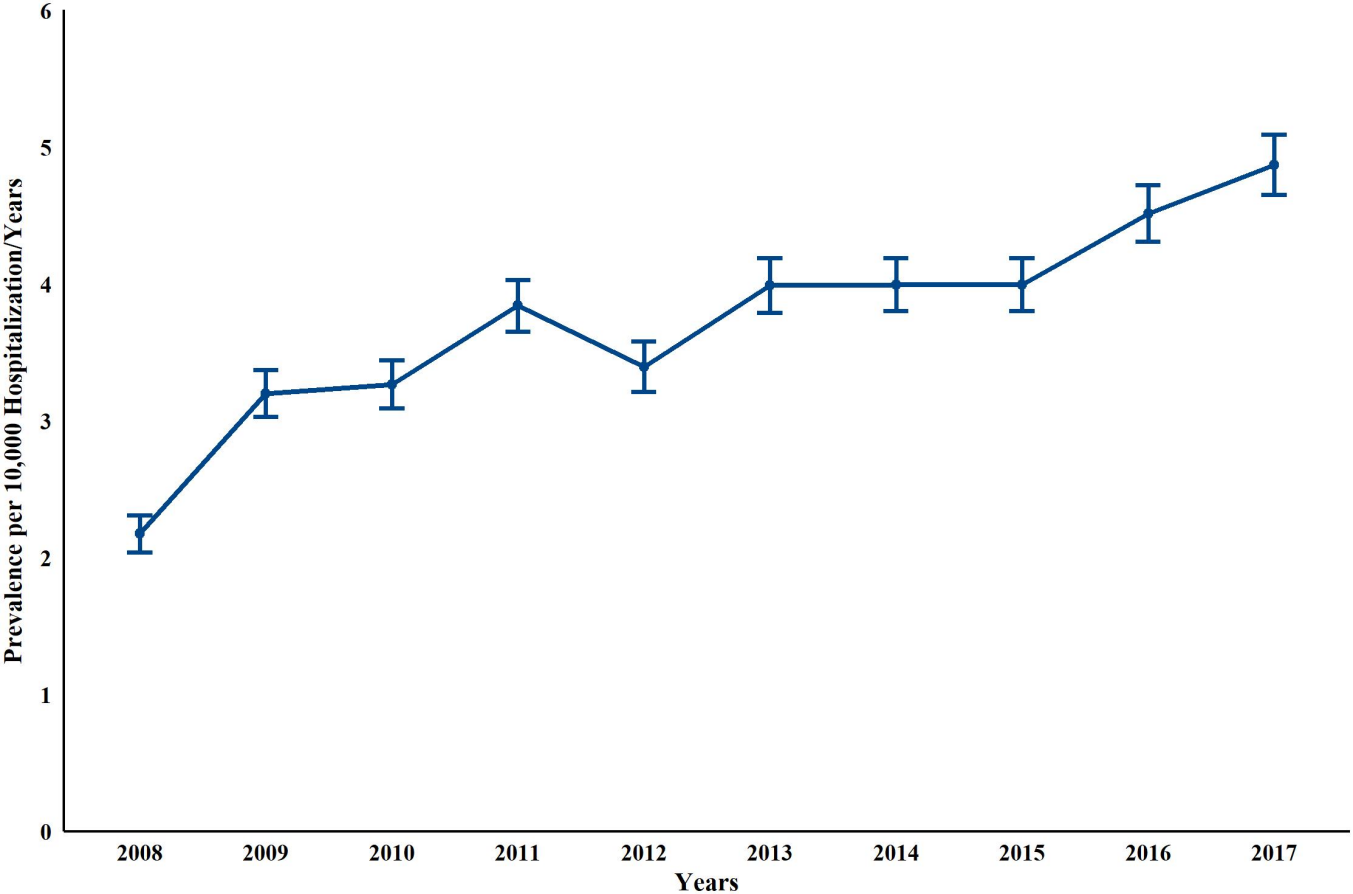
Temporal trends of atrial fibrillation during pregnancy and puerperium. Temporal trend of pregnancy-related atrial fibrillation in the United States from 2008 through 2017. The rates of in-hospital prevalence among patients with atrial fibrillation increased (2.18 per 10,000 in 2008 vs. 4.87 per 10,000 in 2017; *P*_trends_ < 0.001). Error bars represent the 95% confidence interval (95% CI) derived from Normal Approximation method.

The prevalence of AF during pregnancy and puerperium increased from 2.18 (95%CI = 2.04–2.31) per 10,000 hospitalizations in 2008 to 4.87 (95%CI = 4.65–5.09) per 10,000 hospitalizations in 2017 (*P*_trend_ < 0.001) (Figure 2).

**Figure 3:**
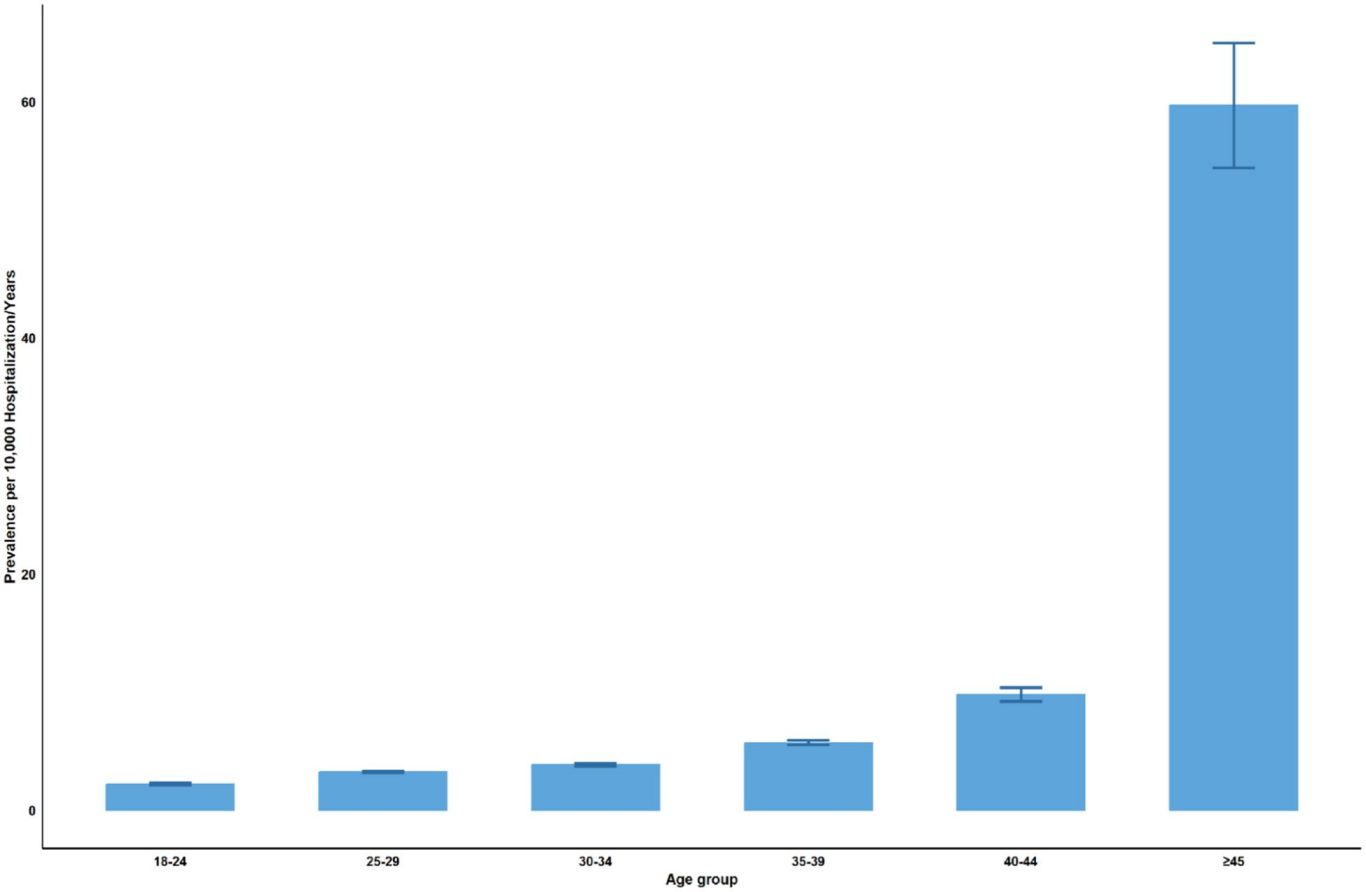
The prevalence of atrial fibrillation increased with age. The prevalence of atrial fibrillation per 100,000 pregnancy-related hospitalizations increased with advancing maternal age. Error bars represents the 95% confidence interval (95% CI) derived from Normal Approximation method.

**Figure 4:**
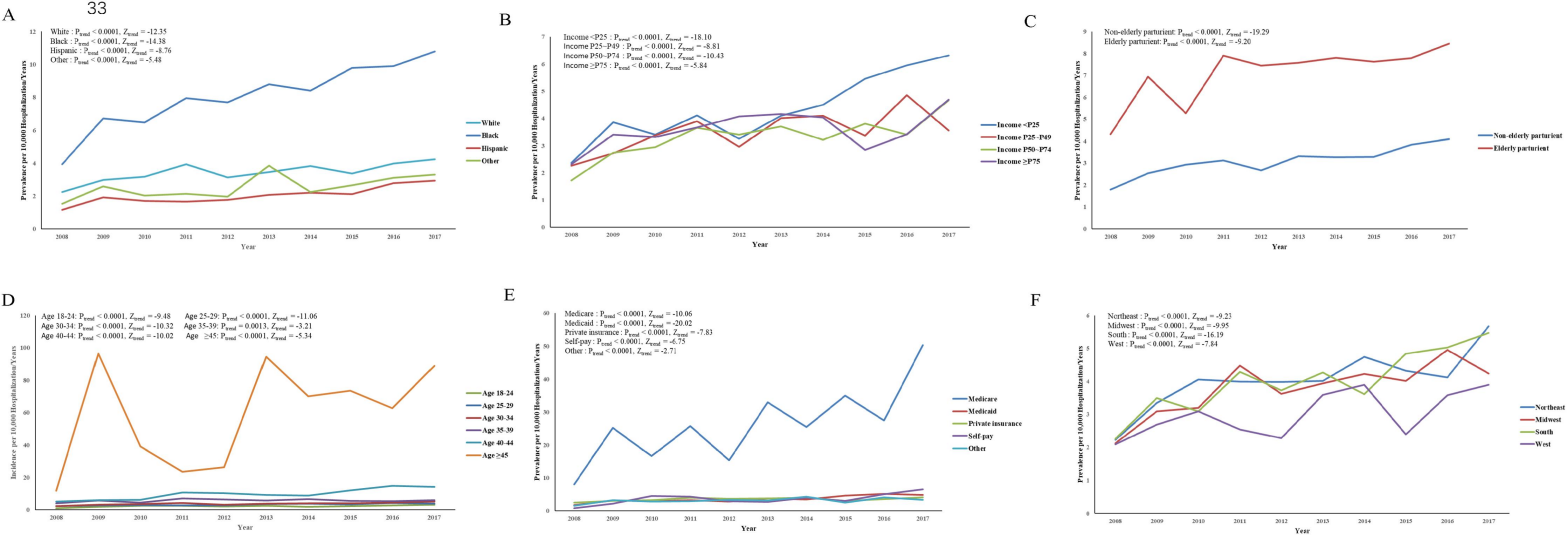
Temporal trends of atrial fibrillation in subgroups. **(A)** Subgroup analyses categorized by race. **(B)** Subgroup analyses categorized by household income. **(C)** Subgroup analyses categorized by old adult parturient. **(D)** Subgroup analyses categorized by age. **(E)** Subgroup analyses categorized by insurance type. **(F)** Subgroup analyses categorized by hospital region exhibited.

The prevalence of AF increased with age, reaching a peak at ages over 45 years (Figure 3). Subgroup analyses based on race, household income, insurance type, age, and hospital region exhibited substantial temporal trend increases among all subgroups, particularly in Black individuals, low-income, medicare insurance, and advanced maternal age (Figure 4).

Maternal patients with AF had a higher prevalence of adverse outcomes compared to patients without AF, including acute heart failure [781/15,162 (5.15%) vs. 19371/40,981,698 (0.05%)], cardiogenic shock [652/15,162 (4.30%) vs. 75,459/40,981,698(0.18%)], acute respiratory failure [359/15,162 (2.37%) vs. 37,395/40,981,698 (0.09%)], acute kidney failure [599/15,162 (3.95%) vs. 34,620/40,981,698 (0.08%)], pulmonary embolism [117/15,162 (0.77%) vs. 33,522/40,981,698 (0.08%)], and acute myocardial infarction [100/15,162 (0.66%)] vs. 3,680/40,981,698 (0.01%)] (all *P* < 0.001). The in-hospital death of patients with AF was 70-fold higher than that of patients without AF [108/15,162 (0.71%) vs. 4,167/40,981,698 (0.01%), *P* < 0.001], (Supplement Table S3). After 1:1 propensity score matching, all covariates achieved adequate balance with standardized differences <0.1 (Supplement Table S10); the adjusted OR for the exposure was 2.14 (95% CI = 1.51–3.06). (Supplement Table S4).

### Secondary outcomes

After controlling for all variables in the models, the following factors were found to be associated with increased AF prevalence: advanced maternal age(OR: 1.87, 95% CI: 1.80–1.94), Black race (OR: 1.70, 95% CI: 1.63–1.77), tobacco abuse (OR: 1.42, 95% CI: 1.36–1.49), obstructive sleep apnea (OR: 2.71, 95% CI: 2.41–3.05), disorders of lipid metabolism (OR: 3.04, 95% CI: 2.75–3.36), thyrotoxicosis (OR: 6.19, 95% CI: 5.52–6.94), chronic pulmonary disease (OR: 1.46, 95% CI: 1.39–1.54), hypertension (OR: 2.38, 95% CI: 2.26–2.50), hypothyroidism (OR: 1.75, 95% CI: 1.64–1.87), congenital heart disease(OR: 9.02, 95%CI: 7.81–10.40), and valvular disease (OR: 8.16, 95% CI: 7.58–8.77) (all *P* < 0.001) (Supplement Table S2).

To avoid the effect of malignancy on mortality, we use data excluding patients with metastatic cancer to explore the relationship between AF and death (Supplement Table S5). Moreover, low-income and Medicare insurance were also significant contributors to the increased prevalence of AF (all *P* < 0.001).

AF (OR: 2.02, 95% CI: 1.56–2.61), age ≥45 (OR: 2.47, 95% CI: 1.89–3.23), metastatic cancer (OR: 50.75, 95% CI: 38.63–66.68), chronic heart failure (OR: 7.42, 95% CI: 6.64–8.29), and pulmonary embolism (OR: 19.80, 95% CI: 17.65–22.22) were all associated with death as shown in Supplement Table S5 (all *P* < 0.001). we assessed its discrimination for in-hospital mortality across subgroups. The c-statistic was only moderate overall (0.67–0.75 across ages, 0.67–0.74 across race/ethnicity), and did not differ significantly between subgroups, with overlapping confidence intervals throughout. Numerically, performance was highest in Black (0.74) and older (0.75) patients (Supplementary Table S1^1^).

There was an increase in mortality with increasing CHA_2_DS_2_-VASc scores. The mortality standardized to patients with CHA_2_DS_2_-VASc score of 1 point was 2.67 (95% CI: 2.49–2.87) in patients with 2 points, 6.29 (95% CI: 5.64–7.00) in patients with 3 points, 12.24 (95% CI: 8.48–13.12) in patients with 4 points, and 17.77 (95% CI: 11.10–28.44) in patients with 5 points (all *P* *<* 0.001). Additionally, after controlling for all variables, AF was also found to be associated with cardiogenic shock (Supplement Table S6), acute respiratory failure (Supplement Table S7), and acute renal failure (Supplement Table S8) during pregnancy- and puerperium-related hospitalizations (all *P* < 0.001).

**Figure 5:**
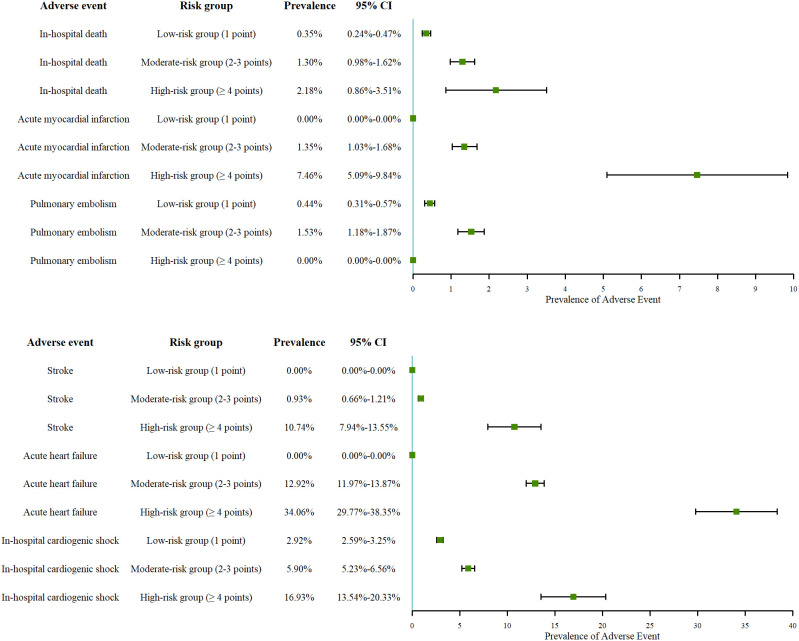
Prevalence of adverse events in different CHA_2_DS_2_-VASc stratification. The mortality rate of AF during pregnancy and puerperium was associated with an increasing CHA_2_DS_2_-VASc score (*P*_trend_ < 0.001), which was 0.35% (95% CI: 0.24%–0.47%) in the low-risk group, 1.30% (95% CI: 0.98%–1.62%) in the moderate-risk group, and 2.18% (95% CI: 0.86%–3.51%) in the high-risk group. The prevalence of stroke, acute myocardial infarction, acute heart failure, and cardiac shock during pregnancy and puerperium was also significantly correlated with the increasing CHA_2_DS_2_-VASc score in AF patients (*P*_trend_ < 0.001).

To further assess the risk stratification of CHA_2_DS_2_-VASc for predicting adverse outcomes of maternal patients with AF, we divided the CHA_2_DS_2_-VASc score into three groups: low-risk group (1 point), moderate-risk group (2–3 points), and high-risk group (≥4 points) (Supplement Table S9). As all patients were female, each automatically received 1 point for sex, making the minimum score 1*.* The results indicated that the increase of CHA_2_DS_2_-VASc stratification was associated with an increased risk of death, stroke, acute myocardial infarction, acute heart failure, and cardiogenic shock (*P* trends all < 0.001). As shown in Table 2 and Figure 5, the mortality was 0.35% (95% CI: 0.24%–0.47%) in the low-risk group, 1.30% (95% CI: 0.98%–1.62%) in the moderate-risk group, and 2.18% (95% CI: 0.86%–3.51%) in the high-risk group, respectively. Nevertheless, compared to the low-risk and moderate-risk groups, the risk of pulmonary embolism was lower in the high CHA_2_DS_2_-VASc scores group (Figure 5).

**Table 2:** Prevalence of adverse events in hospitalizations during pregnancy and puerperium stratified by CHA_2_DS_2_-VASc scores.

Adverse event	Risk group	Prevalence (%)	95% CI (%)	*P* for trend
In-hospital death	Low-risk group (1 point)	0.35	0.24–0.47	<.001
	Moderate-risk group (2–3 points)	1.30	0.98–1.62	
	High-risk group (≥4 points)	2.18	0.86–3.51	
Stroke	Low-risk group (1 point)	0.00	0.00–0.00	<.001
	Moderate-risk group (2–3 points)	0.93	0.66–1.21	
	High-risk group (≥4 points)	10.74	7.94–13.55	
Acute myocardial infarction	Low-risk group (1 point)	0.00	0.00–0.00	<.001
	Moderate-risk group (2–3 points)	1.35	1.03–1.68	
	High-risk group (≥4 points)	7.46	5.09–9.84	
Pulmonary embolism	Low-risk group (1 point)	0.44	0.31–0.57	<.001
	Moderate-risk group (2–3 points)	1.53	1.18–1.87	
	High-risk group (≥4 points)	0.00	0.00–0.00	
Acute heart failure	Low-risk group (1 point)	0.00	0.00–0.00	<.001
	Moderate-risk group (2–3 points)	12.92	11.97–13.87	
	High-risk group (≥4 points)	34.06	29.77–38.35	
Cardiogenic shock	Low-risk group (1 point)	2.92	2.59–3.25	<.001
	Moderate-risk group (2–3 points)	5.90	5.23–6.56	
	High-risk group (≥4 points)	16.93	13.54–20.33	

We also analyzed the impact of AF on the LOS and hospitalization costs (Table 3). The results indicated that AF led to a 0.79-day (95% CI: 0.71–0.89) increase in the LOS and a $16,674(95% CI: 16,059–17,290) rise in hospitalization costs. The cost of hospitalization increased by $541 (95% CI: 537–545) with the progress of the year. In contrast, the LOS decreased by 0.02-day (95% CI: 0.02–0.02) (all *P* < 0.001).

**Table 3:** Association of atrial fibrillation with hospitalization cost and LOS in hospitalizations during pregnancy and puerperium.

Items	Estimate	95% Lower CI	95% Upper CI	*P* Value
Association of atrial fibrillation compared with no atrial fibrillation with each outcome among all participants				
Cost, dollars	16,674	16,059	17,290	<.001
LOS, days	0.79	0.71	0.89	<.001
Association of each outcome with unit increase in year with all patients				
Cost, dollars	541	537	545	<.001
LOS, days	−0.02	−0.02	−0.02	<.001

Obtained from the general linear model with either cost or LOS as the dependent variable and atrial fibrillation as the predictor variable, adjusted for the same covariate assessment of model 1.

## Discussion

In this large national study of 40,996,860 pregnancies, 15,162 (0.037%) cases were diagnosed with AF. We observed a steady increase in the prevalence of maternal AF, rising from 2.18 to 4.87 per 10,000 between 2008 and 2017. The prevalence of in-hospital cardiovascular complications and mortality among maternal patients with AF was significantly higher compared to those without AF. The mortality of patients with AF was 70-fold higher than those without AF. The risk of mortality and adverse cardiovascular events in maternal patients increased with higher CHA_2_DS_2_-VASc scores.

Thakkar et al. (7) used the NIS database to analyze arrhythmias in maternal patients. They found that the prevalence of arrhythmias during delivery hospitalizations increased from 48.67 per 1,000,000 in 2009 to 148.17 per 1,000,000 in 2019, and the most common arrhythmia was supraventricular tachycardia, followed by AF. In contrast, Vaidya et al. (8) reported that AF was the most common arrhythmia in pregnancy, with the proportion increasing from 1.8 to 3.5 per 10,000 patients between 2000 and 2012. Another study conducted by Lee et al. (9) demonstrated an prevalence rate of 61.3 per 100,000 patients for AF in maternal patients. Our study also revealed a national increasing trend of AF during pregnancy and puerperium, from 2.18 to 4.87 per 10,000 between 2008 and 2017. The prevalence of AF varies across studies, possibly due to differences in patient populations, study durations, and healthcare capabilities for diagnosing AF.

AF was reported to be associated with a significant increase in maternal mortality (10). Our study revealed that patients with AF had a mortality rate 70 times higher than those without AF. The rising occurrence of AF during pregnancy and puerperium highlights the importance of identifying the underlying risk factors, which can be modifiable or unmodifiable. Previous epidemiological studies have demonstrated that traditional cardiovascular risk factors are consistently associated with AF (11–13). In the present study, traditional cardiovascular risk factors, including tobacco abuse, lipid metabolism disorders, hypertension, obstructive sleep apnea, hyperthyroidism, and obesity, were significantly associated with the increased risk of AF. A growing amount of evidence suggests that atrial remodeling induced by obstructive sleep apnea and hyperthyroidism contributes to the pathogenesis of AF. Thus, obstructive sleep apnea and hyperthyroidism have been reproducibly identified as risk factors for AF and stroke (14, 15). Identifying and managing the aforementioned risk factors may help reduce the prevalence of AF in the maternal population and improve their prognosis.

Advancing age is a non-modifiable factor in the development of cardiovascular diseases. Advanced maternal age is recognized as an independent risk factor for arrhythmia during delivery hospitalization (9, 16). In our study, we also found a higher prevalence of AF in elderly adult parturients than in non-elderly adult parturients. The prevalence of AF increased with age and peaked at age ≥45. Standardized to patients aged 18–24 years, the prevalence rate of AF was 11.89 in patients aged ≥45 years. However, this age group represented only approximately 0.2% of all pregnancy-related hospitalizations, resulting in a limited denominator that may inflate the prevalence estimate. Therefore, these findings should be interpreted with caution. Larger, population-based studies are warranted to validate this observation. Moreover, our previous report (17) indicated a close association between advanced maternal age and acute high-risk chest pain diseases, including acute myocardial infarction, pulmonary embolism, and aortic dissection. The potential explanation was that the prevalence of cardiovascular risk factors and the prevalence of acquired cardiovascular disease during pregnancy and puerperium both increase with advanced maternal age. Enhancing the monitoring of AF among old adult maternal patients is imperative in the future.

Race is also an unmodifiable factor for AF. In our study, Black individuals had a 2.4-fold higher prevalence of AF compared to white individuals during pregnancy and puerperium. Previous population-based studies have reported that the prevalence of AF is lower in Black populations compared to white populations despite Black individuals having a higher burden of risk factors (18, 19). The conflicting results may be due to the lack of awareness about AF diagnosis and limited access to health care among some Black individuals, leading to delayed diagnosis of AF. The risk of AF during pregnancy and puerperium is also influenced by socio-economic status. The highest prevalence was observed in the lowest median household income category. Insurance status was also associated with AF risk. Previous population-based studies showed that most AF patients were covered by medicare insurance (20, 21). However, pregnant women with medicare insurance account for the lowest proportion in this study but with the highest AF prevalence. The reasons behind this phenomenon may extend beyond the financial aspect. The government should enhance physician awareness regarding AF among old adult and Black maternal patients, particularly those with the lowest median household income and medicare insurance.

We also observed a significant correlation between valvular disease and AF during pregnancy and puerperium. Valvular heart disease has been reported to be linked to the occurrence of arrhythmias (7) and acute high-risk chest pain diseases (17) during pregnancy. A meta-analysis enrolled 7 cohort studies revealed that the prevalence of AF in pregnant women with structural heart disease was 2.2%, compared to 0.3% among those without structural heart disease (22). A study conducted by the Registry of Pregnancy and Cardiac Disease indicated that the prevalence of arrhythmia among pregnant women with valvular heart disease was 2%, and the maternal mortality was 0.6% (23). Therefore, it is imperative to conduct a thorough assessment and closely monitor for the presence of valvular heart disease in pregnant women.

Currently, there is no tool available to predict stroke risk and adverse outcomes in pregnancy. CHA_2_DS_2_-VASc scoring system determines the anticoagulation strategy in patients with non-valvular AF. Recent studies have shown that the validity of the CHA_2_DS_2_-VASc score extends beyond its original application in AF. High CHA_2_DS_2_-VASc scores have been proven to be associated with an increased risk of adverse cardiovascular events and death in several diseases, regardless of the presence or absence of AF. In stable ischemic heart disease, Sanchez et al. (24) reported that a higher CHA_2_DS_2_-VASc score (≥4 points) was associated with increased mortality and stroke risk during long-term follow-up. Goudis et al. (25) summarized the CHA_2_DS_2_-VASc score application in chronic kidney disease, finding that a higher CHA_2_DS_2_-VASc score was associated with an increased risk of cardiovascular and all-cause mortality. However, maternal patients were excluded from the reported CHA_2_DS_2_-VASc applications.

In the present study, we observed an elevated risk of adverse cardio-cerebral outcomes in maternal patients as the CHA_2_DS_2_-VASc stratification increased, especially for those with a CHA_2_DS_2_-VASc ≥4. Maternal patients with AF in the high-risk group (≥4 points) had about six times the risk of death compared to the low-risk group (1 point). A high CHA_2_DS_2_-VASc score was associated with an increased risk of adverse cardiovascular events and death in our study, suggesting the importance of improving management in maternal patients with high CHA_2_DS_2_-VASc scores. Furthermore, as all patients in this cohort were female, a baseline score of 1 was assigned to every individual. This inherent lack of variability may lead to overestimation of absolute predicted risk in the low-risk group and could attenuate the score's overall discriminative capacity. Therefore, the observed associations with CHA₂DS₂-VASc should be interpreted primarily as evidence of a risk gradient rather than as externally calibrated risk estimates. The CHA_2_DS_2_-VASc score may help reduce adverse cardiovascular events and death in the maternal population. Nevertheless, our study found that the risk of pulmonary embolism was lower in the high CHA_2_DS_2_-VASc scores group, compared to the low-risk and moderate-risk groups. This unexpected finding may be partially attributable to the small number of maternal patients with high CHA₂DS₂-VASc scores and AF. In addition, we speculate that these patients—who tend to be older and have more comorbidities—are more likely to undergo planned Cesarean section or early delivery, which could shorten the window of pregnancy-related hypercoagulability and thereby reduce thrombotic events. Further studies with detailed data on delivery timing and procedures are needed to verify this hypothesis.

To our knowledge, the present study was the first to confirm that the mortality and severe cardiovascular events among maternal patients increased in line with the ascending CHA_2_DS_2_-VASc stratification, especially for those maternal patients in the high-risk group. In models 3 and 4, the results of two methods were consistent regarding hospital death, cardiogenic shock, and acute renal failure, thereby strengthening the robustness of the conclusions. The discrepancies observed in acute respiratory failure indicate that the interaction of confounders warrants further investigation. Given that maternal morbidity rises globally, early recognition of high-risk patients becomes more pivotal to preventing major adverse events. In this exploratory analysis, the CHA_2_DS_2_-VASc score showed some ability to discriminate adverse outcomes, suggesting that it may serve as a complementary risk assessment tool in pregnant women with AF; however, its clinical utility requires prospective validation.

### Study limitations

Despite its strengths, the present study has several limitations. First, this study is a non-random and observational design. Second, NIS is a database that relies on the diagnostic code and is prone to coding errors. NIS only provides hospitalization information and does not include data on clinical follow-up. Third, due to limitations of ICD coding, the database cannot differentiate pre-existing AF, paroxysmal AF, or new-onset AF during pregnancy and puerperium. Consequently, we were unable to determine whether the arrhythmia was pre-existing or developed *de novo* in the peripartum period, new-onset AF often accompanies acute life-threatening conditions (e.g., peripartum cardiomyopathy, severe pre-eclampsia) that themselves carry high mortality, profoundly confounding the AF–death association. The observed 70-fold mortality increase likely reflects the composite risk of AF with its acute triggers rather than an independent effect, tending to overestimate the risk attributed to AF. Fourth, limited by the data elements available in the NIS database, crucial information such as medication administration, procedural details, and genetic testing is not captured which may introduce bias into outcome evaluations. Fifth, the NIS database represents the American population. The prevalence of risk factors may differ among various races and regions. Our results may not apply to populations outside of the United States. Finally, the study period spans 2008 to 2017, and practice patterns in obstetric care and thromboprophylaxis have evolved since then. This time lag may limit the applicability of our findings to the current clinical landscape. Despite these limitations, our study utilizes a nationally contemporary database and provides essential information on AF during pregnancy and puerperium. This database illustrates the practices in the real world and minimizes potential selection biases that may arise from specialized centers.

## Conclusions

In this large, nationally representative sample of pregnancy-related hospitalizations, we identified an increasing prevalence of AF between 2008 and 2017. Pregnant women with advanced age (≥45 years), Black individuals, and patients from lowest-income households had a higher likelihood of developing AF. The prevalence of in-hospital cardiovascular complications and mortality among maternal patients with AF was significantly higher than those without AF. In addition, CHA_2_DS_2_-VASc score was associated with mortality in maternal patients with AF. maternal patients with high CHA_2_DS_2_-VASc scores should have increased surveillance. CHA_2_DS_2_-VASc score may be helpful to guide management in maternal patients.

## Data Availability

The datasets presented in this study can be found in online repositories. The names of the repository/repositories and accession number(s) can be found below: the NationwideInpatient Sample (NIS) database.
