# Supplementary material for: Prevalence and outcomes of atrial fibrillation in hospitalizations during pregnancy and puerperium
Source: Front Cardiovasc Med. 2026 Jul 30;13:1814450. doi: 10.3389/fcvm.2026.1814450 (PMC13469427; doi:10.3389/fcvm.2026.1814450)
Supplement: Supplementary file 1 [file Table1.docx]

**Table 1 Comparison of Demographics, Hospital Characteristics, Interventions Between Hospitalization with and without Atrial Fibrillation**

| Variables | All cohort  N = 40,996,860 | Atrial fibrillation | | *P* value |
| --- | --- | --- | --- | --- |
|  |  | Absent  N = 40,981,698 | Present  N = 15,162 |  |
| Age in years at admission | 28.32±12.95 | 28.32±12.94 | 31.16±14.96 | <.001 |
| Age group |  |  |  |  |
| 18~24 | 11922245(29.08) | 11919623(29.09) | 2622(17.29) | <.001 |
| 25~29 | 11936750(29.12) | 11932895(29.12) | 3855(25.43) |  |
| 30~34 | 10693025(26.08) | 10688930(26.08) | 4094(27.00) |  |
| 35~39 | 5208208(12.70) | 5205235(12.70) | 2973(19.61) |  |
| 40~44 | 1154771(2.82) | 1153642(2.82) | 1129(7.45) |  |
| ≥45 | 81861(0.20) | 81372(0.20) | 489(3.22) |  |
| Race |  |  |  |  |
| White | 23412509(57.11) | 23404557(57.11) | 7952(52.45) | <.001 |
| Black | 5595022(13.65) | 5590464(13.64) | 4558(30.06) |  |
| Hispanic | 7869837(19.20) | 7868238(19.20) | 1598(10.54) |  |
| Other | 4119493(10.05) | 4118439(10.05) | 1054(6.95) |  |
| Elderly parturient women* | 6444840(15.72) | 6440250(15.71) | 4590(30.28) | <.001 |
| Admission day is on a weekend | 8005528(19.53) | 8002577(19.53) | 2951(19.47) | 0.8497 |
| Elective admission | 19590492(47.79) | 19585489(47.79) | 5002(32.99) | <.001 |
| Season |  |  |  |  |
| Winter | 9586369(23.38) | 9582773(23.38) | 3596(23.72) | 0.1568 |
| Spring | 9820061(23.95) | 9816344(23.95) | 3717(24.51) |  |
| Summer | 11571708(28.23) | 11567520(28.23) | 4187(27.62) |  |
| Autumn | 10018722(24.44) | 10015061(24.44) | 3661(24.15) |  |
| Control/ownership of hospital |  |  |  |  |
| Government nonfederal | 5252299(12.81) | 5250294(12.81) | 2005(13.22) | <.001 |
| Private not for profit | 30049144(73.30) | 30037648(73.30) | 11496(75.82) |  |
| Private investor owned | 5695418(13.89) | 5693757(13.89) | 1660(10.95) |  |
| Bed size of hospital |  |  |  |  |
| Small | 5414371(13.21) | 5413038(13.21) | 1333(8.79) | <.001 |
| Medium | 11593007(28.28) | 11589461(28.28) | 3546(23.38) |  |
| Large | 23989482(58.52) | 23979199(58.51) | 10283(67.82) |  |
| Teaching status of hospital |  |  |  |  |
| Nonteaching | 18086372(44.12) | 18081975(44.12) | 4397(29.00) | <.001 |
| Teaching | 22910488(55.88) | 22899723(55.88) | 10765(71.00) |  |
| Location status of hospital |  |  |  |  |
| Rural | 4199874(10.24) | 4199103(10.25) | 771(5.08) | <.001 |
| Urban | 36796986(89.76) | 36782595(89.75) | 14391(94.92) |  |
| Region of hospital |  |  |  |  |
| Northeast | 6659219(16.24) | 6656547(16.24) | 2672(17.62) | <.001 |
| Midwest | 8702310(21.23) | 8699055(21.23) | 3255(21.47) |  |
| South | 15716954(38.34) | 15710679(38.34) | 6275(41.39) |  |
| West | 9918377(24.19) | 9915418(24.19) | 2960(19.52) |  |
| Primary expected payer |  |  |  |  |
| Medicare | 326638(0.80) | 325793(0.79) | 845(5.57) | <.001 |
| Medicaid | 17702974(43.18) | 17696619(43.18) | 6355(41.91) |  |
| Private insurance | 20555598(50.14) | 20548425(50.14) | 7172(47.31) |  |
| Self-pay | 1180545(2.88) | 1180141(2.88) | 404(2.66) |  |
| Other | 1231105(3.00) | 1230720(3.00) | 386(2.55) |  |
| Median household income percentile |  |  |  |  |
| <P25 | 11900816(29.03) | 11895682(29.03) | 5134(33.86) | <.001 |
| P25~P49 | 10155987(24.77) | 10152453(24.77) | 3534(23.31) |  |
| P50~P74 | 9982573(24.35) | 9979269(24.35) | 3304(21.79) |  |
| ≥P75 | 8957484(21.85) | 8954294(21.85) | 3190(21.04) |  |
| Tobacco abuse | 3325478(8.11) | 3323154(8.11) | 2324(15.33) | <.001 |
| Alcohol abuse | 67054(0.16) | 66978(0.16) | 75(0.50) | <.001 |
| Drug abuse | 834081(2.03) | 833458(2.03) | 623(4.11) | <.001 |
| Obstructive sleep apnea | 30988(0.08) | 30594(0.07) | 393(2.59) | <.001 |
| Disorders of lipoid metabolism | 81150(0.20) | 80603(0.20) | 547(3.61) | <.001 |
| Nephrotic syndrome | 4922(0.01) | 4918(0.01) | 5(0.03) | 0.0333 |
| Chronic kidney disease | 36433(0.09) | 36024(0.09) | 409(2.70) | <.001 |
| Thyrotoxicosis | 76189(0.19) | 75869(0.19) | 320(2.11) | <.001 |
| Varicose vein of lower limb | 60487(0.15) | 60473(0.15) | 14(0.09) | 0.0887 |
| Acquired immune deficiency syndrome | 10873(0.03) | 10858(0.03) | 15(0.10) | <.001 |
| Deficiency anemias | 3469643(8.46) | 3467270(8.46) | 2373(15.65) | <.001 |
| Rheumatoid arthritis/collagen vascular diseases | 125060(0.31) | 124842(0.30) | 218(1.44) | <.001 |
| Chronic blood loss anemia | 4735410(11.55) | 4732536(11.55) | 2874(18.95) | <.001 |
| Chronic pulmonary disease | 1714902(4.18) | 1713147(4.18) | 1755(11.58) | <.001 |
| Coagulopathy | 733422(1.79) | 732617(1.79) | 805(5.31) | <.001 |
| Depression | 1015765(2.48) | 1014671(2.48) | 1094(7.21) | <.001 |
| Hypertension | 1016069(2.48) | 1013518(2.47) | 2551(16.83) | <.001 |
| Hypothyroidism | 1111936(2.71) | 1110896(2.71) | 1040(6.86) | <.001 |
| Liver disease | 89548(0.22) | 89448(0.22) | 100(0.66) | <.001 |
| Lymphoma | 6817(0.02) | 6812(0.02) | 5(0.03) | 0.1184 |
| Fluid and electrolyte disorders | 495529(1.21) | 493545(1.20) | 1984(13.09) | <.001 |
| Metastatic cancer | 3061(0.01) | 3061(0.01) | 0(0.00) | 0.2872 |
| Other neurological disorders | 357436(0.87) | 356917(0.87) | 519(3.42) | <.001 |
| Obesity | 2568669(6.27) | 2565968(6.26) | 2701(17.82) | <.001 |
| Paralysis | 21771(0.05) | 21717(0.05) | 54(0.36) | <.001 |
| Peripheral vascular disorders | 8407(0.02) | 8326(0.02) | 81(0.53) | <.001 |
| Psychoses | 435042(1.06) | 434639(1.06) | 403(2.65) | <.001 |
| Pulmonary circulation disorders | 19720(0.05) | 19415(0.05) | 305(2.01) | <.001 |
| Solid tumor without metastasis | 11263(0.03) | 11244(0.03) | 19(0.13) | <.001 |
| Peptic ulcer disease excluding bleeding | 2055(0.01) | 2050(0.01) | 5(0.03) | <.001 |
| Valvular disease | 119957(0.29) | 118903(0.29) | 1054(6.95) | <.001 |
| Weight loss | 36860(0.09) | 36658(0.09) | 202(1.33) | <.001 |
| Pre-eclampsia/eclampsia | 1860449(4.54) | 1859614(4.54) | 835(5.50) | <.001 |
| CHA_2_DS_2_-VASc |  |  |  | <.001 |
| Hypertension | 4413999(10.77) | 4410133(10.76) | 3867(25.50) | <.001 |
| Diabetes | 1207027(2.94) | 1205993(2.94) | 1033(6.82) | <.001 |
| Peripheral Vascular Disease | 19725(0.05) | 19395(0.05) | 330(2.18) | <.001 |
| CHF | 52918(0.13) | 51136(0.12) | 1782(11.75) | <.001 |
| Stroke/TIA | 37475(0.09) | 37067(0.09) | 408(2.69) | <.001 |
| CHA_2_DS_2_-VASc Score | 1.00(1.00-1.00) | 1.00(1.00-1.00) | 1.00(1.00-2.00) | <.001 |
| Length of stay days | 2.00(2.00-3.00) | 2.00(2.00-3.00) | 3.00(2.00-4.00) | <.001 |
| Patient disposition |  |  |  | <.001 |
| Routine | 40048372(97.69) | 40034546(97.69) | 13826(91.19) |  |
| Transfer to Short-term Hospital | 42178(0.10) | 42101(0.10) | 77(0.51) |  |
| Transfer to skilled nursing facility or intermediate care facility | 43780(0.11) | 43596(0.11) | 184(1.21) |  |
| Home Health Care | 741324(1.81) | 740568(1.81) | 757(4.99) |  |
| Against Medical Advice | 116284(0.28) | 116073(0.28) | 210(1.39) |  |
| In-hospital death | 4274(0.01) | 4167(0.01) | 108(0.71) |  |
| Discharge alive destination unknown | 647(0.00) | 647(0.00) | 0(0.00) |  |
| *Elderly parturient women were defined as female during pregnancy or puerperium whose age older than 35-year-old.  Causing the weight of observation may not be integer, the count of each group may less or more than the total of all group after rounding off. CHF,Chronic heart failure;TIA,Transient ischemic attack. | | | | |
