# Supplementary material for: Prevalence and outcomes of atrial fibrillation in hospitalizations during pregnancy and puerperium
Source: Front Cardiovasc Med. 2026 Jul 30;13:1814450. doi: 10.3389/fcvm.2026.1814450 (PMC13469427; doi:10.3389/fcvm.2026.1814450)
Supplement: Supplementary file 2 [file Table10.docx]

**Supplement Table 10 Comparison of Standardized Differences Before and After Propensity Score Matching**

| Variables | Before PSM | After PSM |
| --- | --- | --- |
|  |  |  |
| Age group |  |  |
| 18~24 | Ref | Ref |
| 25~29 | <0.01 | 0.01 |
| 30~34 | 0.02 | <0.01 |
| 35~39 | 0.19 | <0.01 |
| 40~44 | 0.21 | <0.01 |
| ≥45 | 0.23 | 0.02 |
| Race |  |  |
| White | Ref | Ref |
| Black | 0.41 | <0.01 |
| Hispanic | <0.01 | 0.05 |
| Other | <0.01 | 0.03 |
| Admission day is on a weekend | <0.01 | <0.01 |
| Elective admission | <0.01 | 0.02 |
| Season |  |  |
| Winter | Ref | Ref |
| Spring | 0.01 | <0.01 |
| Summer | <0.01 | 0.04 |
| Autumn | <0.01 | 0.02 |
| Control/ownership of hospital |  |  |
| Government nonfederal | Ref | Ref |
| Private not for profit | 0.06 | <0.01 |
| Private investor owned | <0.01 | <0.01 |
| Bed size of hospital |  |  |
| Small | Ref | Ref |
| Medium | <0.01 | <0.01 |
| Large | 0.19 | 0.02 |
| Teaching status of hospital |  |  |
| Nonteaching | Ref | Ref |
| Teaching | 0.32 | <0.01 |
| Location status of hospital |  |  |
| Rural | Ref | Ref |
| Urban | 0.19 | 0.01 |
| Region of hospital |  |  |
| Northeast | Ref | Ref |
| Midwest | 0.01 | 0.01 |
| South | 0.06 | <0.01 |
| West | <0.01 | 0.02 |
| Primary expected payer |  |  |
| Medicare | Ref | Ref |
| Medicaid | <0.01 | 0.01 |
| Private insurance | <0.01 | <0.01 |
| Self-pay | <0.01 | 0.03 |
| Other | <0.01 | <0.01 |
| Median household income percentile |  |  |
| <P25 | Ref | Ref |
| P25~P49 | <0.01 | <0.01 |
| P50~P74 | <0.01 | 0.02 |
| ≥P75 | <0.01 | <0.01 |
| Tobacco abuse | 0.23 | <0.01 |
| Alcohol abuse | 0.06 | <0.01 |
| Drug abuse | 0.12 | 0.01 |
| Obstructive sleep apnea | 0.22 | 0.04 |
| Disorders of lipoid metabolism | 0.25 | 0.03 |
| Nephrotic syndrome | 0.01 | <0.01 |
| Chronic kidney disease | 0.22 | 0.05 |
| Thyrotoxicosis | 0.18 | <0.01 |
| Varicose vein of lower limb | <0.01 | 0.01 |
| Acquired immune deficiency syndrome | 0.03 | 0.02 |
| Deficiency anemias | 0.22 | <0.01 |
| Rheumatoid arthritis/collagen vascular diseases | 0.12 | 0.02 |
| Chronic blood loss anemia | 0.21 | <0.01 |
| Chronic pulmonary disease | 0.28 | <0.01 |
| Coagulopathy | 0.19 | <0.01 |
| Depression | 0.22 | <0.01 |
| Hypothyroidism | 0.2 | <0.01 |
| Liver disease | 0.07 | <0.01 |
| Lymphoma | 0.01 | 0.02 |
| Fluid and electrolyte disorders | 0.47 | <0.01 |
| Metastatic cancer | <0.01 | <0.01 |
| Other neurological disorders | 0.18 | <0.01 |
| Obesity | 0.36 | <0.01 |
| Paralysis | 0.07 | 0.03 |
| Peripheral vascular disorders | 0.12 | <0.01 |
| Pulmonary circulation disorders | 0.19 | <0.01 |
| Solid tumor without metastasis | 0.04 | <0.01 |
| Peptic ulcer disease excluding bleeding | 0.02 | 0.02 |
| Valvular disease | 0.36 | <0.01 |
| Weight loss | 0.15 | 0.02 |
| Pre-eclampsia/eclampsia | 0.17 | 0.02 |
| CHA2DS2-VASc |  |  |
| Hypertension | 0.39 | <0.01 |
| Diabets | 0.18 | <0.01 |
| Perpheral Vascular Disease | 0.2 | 0.08 |
| CHF | 0.51 | 0.06 |
| Stroke/TIA | 0.22 | 0.02 |
