# Supplementary material for: Prevalence and outcomes of atrial fibrillation in hospitalizations during pregnancy and puerperium
Source: Front Cardiovasc Med. 2026 Jul 30;13:1814450. doi: 10.3389/fcvm.2026.1814450 (PMC13469427; doi:10.3389/fcvm.2026.1814450)
Supplement: Supplementary file 3 [file Table11.docx]

**Supplement Table 11 Predictive Performance of CHA₂DS₂‑VASc Score for In‑Hospital Mortality Across Subgroups**

| Subgroups | AUC | 95%CI |
| --- | --- | --- |
|  |  |  |
| Age group |  |  |
| 18~24 | 0.6678 | 0.6325-0.7031 |
| 25~29 | 0.6975 | 0.6632-0.7317 |
| 30~34 | 0.6967 | 0.6631-0.7303 |
| 35~39 | 0.7137 | 0.6699-0.7576 |
| 40~44 | 0.7215 | 0.6554-0.7876 |
| ≥45 | 0.7518 | 0.6112-0.8923 |
| Race |  |  |
| White | 0.6686 | 0.6428-0.6944 |
| Black | 0.7383 | 0.7069-0.7697 |
| Hispanic | 0.6950 | 0.6497-0.7402 |
| Other | 0.6864 | 0.6346-0.7382 |
