# Supplementary material for: Prevalence and outcomes of atrial fibrillation in hospitalizations during pregnancy and puerperium
Source: Front Cardiovasc Med. 2026 Jul 30;13:1814450. doi: 10.3389/fcvm.2026.1814450 (PMC13469427; doi:10.3389/fcvm.2026.1814450)
Supplement: Supplementary file 4 [file Table2.docx]

**Table 2** **Prevalence of adverse events in Hospitalizations During Pregnancy and Puerperium stratified by CHA_2_DS_2_-VASc scores**

| Adverse event | Risk group | Prevalence (%) | 95% CI (%) | *P* for trend |
| --- | --- | --- | --- | --- |
| In-hospital death | Low-risk group (1 point) | 0.35 | 0.24-0.47 | < .001 |
|  | Moderate-risk group (2-3 points) | 1.30 | 0.98-1.62 |  |
|  | High-risk group (≥4 points) | 2.18 | 0.86-3.51 |  |
| Stroke | Low-risk group (1 point) | 0.00 | 0.00-0.00 | < .001 |
|  | Moderate-risk group (2-3 points) | 0.93 | 0.66-1.21 |  |
|  | High-risk group (≥4 points) | 10.74 | 7.94-13.55 |  |
| Acute myocardial infarction | Low-risk group (1 point) | 0.00 | 0.00-0.00 | < .001 |
|  | Moderate-risk group (2-3 points) | 1.35 | 1.03-1.68 |  |
|  | High-risk group (≥4 points) | 7.46 | 5.09-9.84 |  |
| Pulmonary embolism | Low-risk group (1 point) | 0.44 | 0.31-0.57 | < .001 |
|  | Moderate-risk group (2-3 points) | 1.53 | 1.18-1.87 |  |
|  | High-risk group (≥4 points) | 0.00 | 0.00-0.00 |  |
| Acute heart failure | Low-risk group (1 point) | 0.00 | 0.00-0.00 | < .001 |
|  | Moderate-risk group (2-3 points) | 12.92 | 11.97-13.87 |  |
|  | High-risk group (≥4 points) | 34.06 | 29.77-38.35 |  |
| Cardiogenic shock | Low-risk group (1 point) | 2.92 | 2.59-3.25 | < .001 |
|  | Moderate-risk group (2-3 points) | 5.90 | 5.23-6.56 |  |
|  | High-risk group (≥4 points) | 16.93 | 13.54-20.33 |  |
