# Supplementary material for: Prevalence and outcomes of atrial fibrillation in hospitalizations during pregnancy and puerperium
Source: Front Cardiovasc Med. 2026 Jul 30;13:1814450. doi: 10.3389/fcvm.2026.1814450 (PMC13469427; doi:10.3389/fcvm.2026.1814450)
Supplement: Supplementary file 5 [file Table3.docx]

**Table 3 Association of Atrial Fibrillation with Hospitalization Cost and LOS in Hospitalizations During Pregnancy and Puerperium**

| Items | Estimate | 95% Lower CI | 95% Upper CI | *P* Value |
| --- | --- | --- | --- | --- |
| Association of atrial fibrillation compared with no atrial fibrillation with each outcome among all participants |  |  |  |  |
| Cost, dollars | 16674 | 16059 | 17290 | <.001 |
| LOS, days | 0.79 | 0.71 | 0.89 | <.001 |
| Association of each outcome with unit increase in year with all patients |  |  |  |  |
| Cost, dollars | 541 | 537 | 545 | <.001 |
| LOS, days | -0.02 | -0.02 | -0.02 | <.001 |

Obtained from the general linear model with either cost or LOS as the dependent variable and atrial fibrillation as the predictor variable, adjusted for the same covariate assessment of model 1.
