# Supplementary material for: Prevalence and outcomes of atrial fibrillation in hospitalizations during pregnancy and puerperium
Source: Front Cardiovasc Med. 2026 Jul 30;13:1814450. doi: 10.3389/fcvm.2026.1814450 (PMC13469427; doi:10.3389/fcvm.2026.1814450)
Supplement: Supplementary file 6 [file Table4.docx]

**Supplement Table 4 Association of In-hospital Death with Atrial Fibrillation and Other Clinical Factors in Hospitalization During Pregnancy and Puerperium**

| Variables | Unadjusted Model | | Multivariate Model 1 | | Multivariate Model 2 | | Multivariate Model 3 | | Multivariate Model 4 | |
| --- | --- | --- | --- | --- | --- | --- | --- | --- | --- | --- |
|  | OR (95% CI) | *P* value | OR (95% CI) | *P* value | OR (95% CI) | *P* value | OR(95% CI) | *P* value | OR(95% CI) | *P* value |
| Atrial fibrillation | 70.40(58.11-85.29) | <.001 | 2.02(1.56-2.61) | <.001 | 3.43(2.68-4.39) | <.001 | 1.95(1.50-2.52) | < .001 | 2.14(1.51-3.06) | < 0.001 |
| Age group |  |  |  |  |  |  |  |  |  |  |
| 18~24-25~29 | 1.08(0.99-1.18) | 0.0813 | 1.10(1.00-1.20) | 0.0408 |  |  | 1.10(1.00-1.20) | 0.0447 |  |  |
| 18~24-30~34 | 1.26(1.15-1.37) | <.001 | 1.27(1.16-1.39) | <.001 |  |  | 1.26(1.15-1.38) | < .001 |  |  |
| 18~24-35~39 | 1.58(1.43-1.74) | <.001 | 1.37(1.23-1.52) | <.001 |  |  | 1.37(1.23-1.52) | < .001 |  |  |
| 18~24-40~44 | 2.96(2.60-3.38) | <.001 | 1.96(1.70-2.27) | <.001 |  |  | 1.95(1.69-2.26) | < .001 |  |  |
| 18~24-≥45 | 12.99(10.46-16.14) | <.001 | 2.47(1.89-3.23) | <.001 |  |  | 2.25(1.71-2.96) | < .001 |  |  |
| Race |  |  |  |  |  |  |  |  |  |  |
| White-Black | 2.82(2.62-3.02) | <.001 | 1.36(1.26-1.48) | <.001 | 1.33(1.23-1.44) | <.001 | 0.80(0.73-0.88) | < .001 |  |  |
| White-Hispanic | 0.96(0.88-1.06) | 0.4230 | 0.87(0.79-0.97) | 0.0081 | 0.85(0.77-0.94) | 0.0013 | 1.24(1.11-1.37) | < .001 |  |  |
| White-Other | 1.42(1.29-1.57) | <.001 | 1.33(1.19-1.48) | <.001 | 1.32(1.18-1.47) | <.001 | 1.55(1.39-1.73) | < .001 |  |  |
| Season |  |  |  |  |  |  |  |  |  |  |
| Winter-Spring | 0.93(0.85-1.02) | 0.1137 |  |  |  |  | 1.03(0.96-1.12) | 0.4145 |  |  |
| Winter-Summer | 1.03(0.95-1.12) | 0.4203 |  |  |  |  | 0.94(0.87-1.02) | 0.1319 |  |  |
| Winter-Autumn | 0.92(0.84-1.00) | 0.0506 |  |  |  |  | 1.26(1.07-1.48) | 0.0047 |  |  |
| Admission day is on a weekend | 1.25(1.16-1.34) | <.001 |  |  |  |  | 1.11(1.03-1.20) | 0.0066 |  |  |
| Primary expected pay |  |  |  |  |  |  |  |  |  |  |
| Medicare-Medicaid | 0.22(0.19-0.26) | <.001 | 1.27(1.06-1.52) | 0.0088 | 1.04(0.87-1.23) | 0.6669 | 0.67(0.62-0.72) | < .001 |  |  |
| Medicare-Private insurance | 0.12(0.10-0.14) | <.001 | 0.87(0.72-1.04) | 0.1220 | 0.74(0.62-0.89) | 0.0010 | 1.63(1.42-1.87) | < .001 |  |  |
| Medicare-Self-pay | 0.36(0.30-0.44) | <.001 | 2.05(1.66-2.55) | <.001 | 1.79(1.45-2.21) | <.001 | 0.90(0.75-1.08) | 0.2533 |  |  |
| Medicare-Other | 0.19(0.15-0.24) | <.001 | 1.16(0.90-1.49) | 0.2491 | 0.98(0.77-1.25) | 0.8795 | 1.47(0.78-2.76) | 0.2349 |  |  |
| Median household income |  |  |  |  |  |  |  |  |  |  |
| <25-25~49 | 0.85(0.79-0.91) | <.001 | 1.15(1.06-1.24) | <.001 | 1.14(1.06-1.24) | 0.0010 | 0.80(0.73-0.87) | < .001 |  |  |
| <25-50~74 | 0.61(0.56-0.67) | <.001 | 0.89(0.81-0.97) | 0.0094 | 0.90(0.82-0.99) | 0.0225 | 0.71(0.64-0.78) | < .001 |  |  |
| <25-≥75 | 0.47(0.42-0.51) | <.001 | 0.77(0.70-0.86) | <.001 | 0.81(0.73-0.90) | <.001 |  |  |  |  |
| Elective admission | 0.27(0.25-0.30) | <.001 | 0.48(0.45-0.52) | <.001 | 0.47(0.44-0.51) | <.001 | 0.48(0.44-0.52) | < .001 |  |  |
| Control/ownership of hospital |  |  |  |  |  |  |  |  |  |  |
| Government non-federal- Private not for profit | 0.77(0.71-0.84) | <.001 |  |  |  |  | 1.15(1.04-1.27) | 0.0059 |  |  |
| Government non-federal- Private investor owned | 0.72(0.64-0.81) | <.001 |  |  |  |  | 1.92(1.35-2.73) | < .001 |  |  |
| Bed size of hospital |  |  |  |  |  |  |  |  |  |  |
| Small-Medium | 2.25(1.95-2.60) | <.001 | 1.88(1.62-2.18) | <.001 | 1.89(1.63-2.19) | <.001 | 1.22(1.14-1.31) | < .001 |  |  |
| Small-Large | 3.01(2.62-3.44) | <.001 | 2.01(1.75-2.31) | <.001 | 2.06(1.79-2.36) | <.001 |  |  |  |  |
| Teaching hospital | 2.21(2.07-2.37) | <.001 | 1.39(1.29-1.49) | <.001 | 1.41(1.31-1.52) | <.001 | 1.34(1.24-1.45) | < .001 |  |  |
| Urban hospital | 2.36(2.04-2.72) | <.001 |  |  |  |  | 1.31(1.12-1.53) | < .001 |  |  |
| Region of hospital |  |  |  |  |  |  |  |  |  |  |
| Northeast-Midwest | 0.91(0.82-1.02) | 0.0943 | 0.82(0.74-0.92) | <.001 | 0.81(0.72-0.90) | 0.001 | 0.72(0.64-0.80) | < .001 |  |  |
| Northeast-South | 1.36(1.24-1.48) | <.0001 | 1.11(1.01-1.22) | 0.0334 | 1.07(0.97-1.17) | 0.1639 | 1.06(0.97-1.17) | 0.2136 |  |  |
| Northeast-West | 0.85(0.76-0.94) | 0.0014 | 0.86(0.77-0.96) | 0.0065 | 0.86(0.77-0.96) | 0.0064 | 0.80(0.72-0.89) | < .001 |  |  |
| Tobacco abuse | 1.42(1.29-1.56) | <.001 | 0.74(0.67-0.83) | <.001 | 0.76(0.68-0.84) | <.001 | 0.72(0.64-0.80) | < .001 |  |  |
| Alcohol abuse | 5.10(3.66-7.09) | <.001 |  |  |  |  | 0.82(0.56-1.20) | 0.3032 |  |  |
| Drug abuse | 3.87(3.45-4.34) | <.001 | 1.57(1.37-1.80) | <.001 | 1.52(1.32-1.73) | <.001 | 1.57(1.37-1.80) | < .001 |  |  |
| Obstructive sleep apnea | 10.60(7.56-14.86) | <.001 | 0.44(0.30-0.65) | <.001 |  |  | 0.46(0.32-0.68) | < .001 |  |  |
| Disorders of lipoid metabolism | 7.35(5.72-9.46) | <.001 | 0.46(0.34-0.62) | <.001 |  |  | 0.44(0.33-0.60) | < .001 |  |  |
| Nephrotic syndrome | 20.51(11.18-37.63) | <.001 |  |  |  |  | 1.03(0.54-1.98) | 0.9189 |  |  |
| Chronic kidney disease | 49.60(42.71-57.62) | <.001 | 1.51(1.26-1.82) | <.001 | 1.70(1.42-2.03) | <.001 | 1.53(1.27-1.85) | < .001 |  |  |
| Thyrotoxicosis | 2.66(1.73-4.08) | <.001 |  |  |  |  | 1.10(0.71-1.72) | 0.6726 |  |  |
| Varicose vein of lower limb | 0.00(0.00-9.188E45) | 0.8656 |  |  |  |  |  |  |  |  |
| Acquired immune deficiency syndrome | 40.46(30.17-54.27) | <.001 | 10.73(7.70-14.95) | <.001 | 9.97(7.13-13.96) | <.001 | 11.12(7.97-15.52) | < .001 |  |  |
| Deficiency anemias | 1.90(1.74-2.06) | <.001 |  |  |  |  | 0.86(0.77-0.96) | 0.0071 |  |  |
| Rheumatoid arthritis/collagen vascular diseases | 6.48(5.22-8.06) | <.001 |  |  |  |  | 1.09(0.86-1.39) | 0.4826 |  |  |
| Chronic blood loss anemia | 1.91(1.77-2.06) | <.001 | 0.61(0.56-0.67) | <.001 | 0.59(0.54-0.64) | <.001 | 0.69(0.62-0.76) | < .001 |  |  |
| Chronic pulmonary disease | 2.27(2.04-2.52) | <.001 |  |  |  |  | 1.08(0.96-1.21) | 0.186 |  |  |
| Coagulopathy | 29.59(27.79-31.51) | <.001 | 11.56(10.75-12.43) | <.001 | 10.99(10.22-11.82) | <.001 | 11.54(10.73-12.42) | < .001 |  |  |
| Depression | 1.79(1.54-2.07) | <.001 | 0.64(0.54-0.75) | <.001 | 0.65(0.56-0.77) | <.001 | 0.62(0.53-0.73) | < .001 |  |  |
| Hypothyroidism | 1.41(1.21-1.65) | <.001 |  |  |  |  | 0.91(0.76-1.07) | 0.2572 |  |  |
| Liver disease | 13.68(11.44-16.35) | <.001 | 2.27(1.86-2.77) | <.001 | 2.38(1.95-2.90) | <.001 | 2.20(1.80-2.69) | < .001 |  |  |
| Lymphoma | 13.54(7.18-25.53) | <.001 | 4.34(2.24-8.42) | <.001 | 4.81(2.50-9.25) | <.001 | 4.16(2.14-8.08) | < .001 |  |  |
| Fluid and electrolyte disorders | 67.24(63.30-71.42) | <.001 | 15.25(14.14-16.45) | <.001 | 16.16(15.01-17.40) | <.001 | 15.4(14.28-16.62) | < .001 |  |  |
| Metastatic cancer | 280.46(225.67-348.55 | <.001 | 50.75(38.63-66.68) | <.001 | 51.20(38.95-67.31) | <.001 | 50.56(38.47-66.44) | < .001 |  |  |
| Other neurological disorders | 23.16(21.38-25.09) | <.001 | 7.32(6.66-8.05) | <.001 | 6.48(5.90-7.12) | <.001 | 7.16(6.5-7.88) | < .001 |  |  |
| Obesity | 2.31(2.12-2.53) | <.001 | 1.17(1.06-1.30) | 0.0014 |  |  | 1.23(1.12-1.36) | < .001 |  |  |
| Paralysis | 56.95(47.64-68.08) | <.001 | 3.56(2.86-4.42) | <.001 | 2.78(2.24-3.46) | <.001 | 2.70(2.14-3.40) | < .001 |  |  |
| Psychoses | 1.97(1.60-2.43) | <.001 | 0.60(0.48-0.76) | <.001 | 0.57(0.46-0.72) | <.001 | 0.60.(0.47-0.75) | < .001 |  |  |
| Pulmonary circulation disorders | 121.19(106.12-138.40 | <.001 | 1.44(1.21-1.72) | <.001 | 1.57(1.31-1.88) | <.001 | 1.45(1.21-1.74) | < .001 |  |  |
| Solid tumor without metastasis | 29.47(21.04-41.27) | <.001 | 11.20(7.69-16.31) | <.001 | 11.42(7.81-16.69) | <.001 | 11.29(7.75-16.46) | < .001 |  |  |
| Peptic ulcer disease excluding bleeding | 47.10(25.30-87.68) | <.001 | 3.77(1.87-7.61) | <.001 | 4.50(2.25-8.98) | <.001 | 3.69(1.83-7.44) | < .001 |  |  |
| Valvular disease | 14.17(12.16-16.52) | <.001 |  |  | 1.56(1.30-1.88) | <.001 | 1.05(0.87-1.27) | 0.5949 |  |  |
| Weight loss | 63.95(55.99-73.04) | <.001 | 2.07(1.76-2.43) | <.001 | 2.41(2.06-2.82) | <.001 | 2.01(1.71-2.36) | < .001 |  |  |
| Pulmonary embolism | 207.21(190.09-225.88 | <.001 | 19.80(17.65-22.22) | <.001 | 21.35(19.03-23.96) | <.001 | 19.76(17.6-22.18) | < .001 |  |  |
| Congenital heart disease | 17.75(13.02-24.21) | <.001 | 1.92(1.29-2.86) | 0.0014 | 2.18(1.49-3.20) | <.001 |  |  |  |  |
| CHA_2_DS_2_-VASc |  |  |  |  |  |  |  |  |  |  |
| Hypertension | 4.18(3.92-4.45) | <.001 | 1.71(1.59-1.84) | <.001 |  |  | 1.77(1.64-1.90) | < .001 |  |  |
| Diabetes | 3.04(2.73-3.38) | <.001 | 1.24(1.09-1.39) | <.001 |  |  | 1.26(1.11-1.42) | < .001 |  |  |
| Peripheral Vascular Disease | 164.31(146.21-184.64 | <.001 | 5.80(4.99-6.74) | <.001 |  |  | 5.26(4.50-6.15) | < .001 |  |  |
| CHF | 152.07(140.13-165.01 | <.001 | 7.42(6.64-8.29) | <.001 |  |  | 7.43(6.63-8.33) | < .001 |  |  |
| Stroke/TIA | 28.81(23.82-34.84) | <.001 |  |  |  |  | 1.88(1.58-2.23) | < .001 |  |  |
| 1 point-2 points | 5.07(4.74-5.42) | <.001 |  |  | 2.67(2.49-2.87) | <.001 | 1.95(1.50-2.52) | < .001 |  |  |
| 1 point-3 points | 25.50(23.22-28.00) | <.001 |  |  | 6.29(5.64-7.00) | <.001 |  |  |  |  |
| 1 point-4 points | 228.64(191.94-272.35 | <.001 |  |  | 12.24(9.90-15.14) | <.001 | 1.10(1.00-1.20) | 0.0447 |  |  |
| 1 point-5 points | 443.95(296.60-664.52 | <.001 |  |  | 17.77(11.10-28.44) | <.001 | 1.26(1.15-1.38) | < .001 |  |  |
| 1 point-6 points | 0.01(0.00-7.14E121) | 0.9745 |  |  | 0.00(0.00-4.408E55) | 0.9277 | 1.37(1.23-1.52) | < .001 |  |  |

CHF,Chronic heart failure;TIA,Transient ischemic attack.
