# Supplementary material for: Prevalence and outcomes of atrial fibrillation in hospitalizations during pregnancy and puerperium
Source: Front Cardiovasc Med. 2026 Jul 30;13:1814450. doi: 10.3389/fcvm.2026.1814450 (PMC13469427; doi:10.3389/fcvm.2026.1814450)
Supplement: Supplementary file 7 [file Table5.docx]

**Supplement Table 5 Association of In-hospital Death with Atrial Fibrillation and Other Clinical Factors Excluding Metastatic Cancer in Hospitalization During Pregnancy and Puerperium**

| Variables | Unadjusted Model | | Multivariate Model 1 | | Multivariate Model 2 | |
| --- | --- | --- | --- | --- | --- | --- |
|  | OR (95% CI) | *P* value | OR (95% CI) | *P* value | OR (95% CI) | *P* value |
| Atrial fibrillation | 71.86(59.31-87.07) | <.001 | 2.04(1.57-2.64) | <.001 | 3.38(2.64-4.33) | <.001 |
| Age group |  |  |  |  |  |  |
| 18~24-25~29 | 1.06(0.98-1.16) | 0.1572 | 1.10(1.00-1.20) | 0.0395 |  |  |
| 18~24-30~34 | 1.23(1.13-1.34) | <.001 | 1.27(1.16-1.40) | <.001 |  |  |
| 18~24-35~39 | 1.55(1.41-1.71) | <.001 | 1.38(1.24-1.54) | <.001 |  |  |
| 18~24-40~44 | 2.87(2.51-3.28) | <.001 | 1.94(1.68-2.25) | <.001 |  |  |
| 18~24-≥45 | 12.33(9.87-15.40) | <.001 | 2.26(1.71-2.99) | <.001 |  |  |
| Race |  |  |  |  |  |  |
| White-Black | 2.86(2.66-3.07) | <.001 | 1.37(1.26-1.49) | <.001 | 1.33(1.23-1.45) | <.001 |
| White-Hispanic | 0.92(0.84-1.01) | 0.0945 | 0.83(0.75-0.92) | <.001 | 0.81(0.73-0.90) | <.001 |
| White-Other | 1.42(1.28-1.57) | <.001 | 1.32(1.18-1.47) | <.001 | 1.32(1.18-1.47) | <.001 |
| Season |  |  |  |  |  |  |
| Winter-Spring | 0.92(0.84-1.01) | 0.0675 |  |  |  |  |
| Winter-Summer | 1.03(0.95-1.12) | 0.4863 |  |  |  |  |
| Winter-Autumn | 0.91(0.83-1.00) | 0.0388 |  |  |  |  |
| Admission day is on a weekend | 1.27(1.18-1.36) | <.001 | 1.13(1.05-1.22) | 0.0017 | 1.14(1.06-1.23) | 0.006 |
| Primary expected pay |  |  |  |  |  |  |
| Medicare-Medicaid | 0.23(0.19-0.26) | <.001 | 1.24(1.03-1.48) | 0.0200 | 1.02(0.86-1.21) | 0.8389 |
| Medicare-Private insurance | 0.12(0.10-0.14) | <.001 | 0.84(0.70-1.01) | 0.0611 | 0.73(0.61-0.87) | <.001 |
| Medicare-Self-pay | 0.37(0.31-0.45) | <.001 | 2.01(1.62-2.50) | <.001 | 1.77(1.43-2.19) | <.001 |
| Medicare-Other | 0.19(0.15-0.23) | <.001 | 1.13(0.88-1.46) | 0.3349 | 0.97(0.76-1.24) | 0.8036 |
| Median household income |  |  |  |  |  |  |
| <25-25~49 | 0.84(0.77-0.90) | <.001 | 1.13(1.04-1.22) | 0.0035 | 1.13(1.04-1.22) | 0.0033 |
| <25-50~74 | 0.59(0.54-0.64) | <.001 | 0.87(0.80-0.96) | 0.0042 | 0.89(0.81-0.98) | 0.0127 |
| <25-≥75 | 0.46(0.42-0.50) | <.001 | 0.76(0.69-0.85) | <.001 | 0.80(0.72-0.89) | <.001 |
| Elective admission | 0.27(0.25-0.30) | <.001 | 0.48(0.45-0.52) | <.001 | 0.47(0.44-0.51) | <.001 |
| Control/ownership of hospital |  |  |  |  |  |  |
| Government non-federal- Private not for profit | 0.79(0.72-0.85) | <.001 |  |  |  |  |
| Government non-federal- Private investor owned | 0.73(0.65-0.82) | <.001 |  |  |  |  |
| Bed size of hospital |  |  |  |  |  |  |
| Small-Medium | 2.27(1.96-2.63) | <.001 | 1.90(1.64-2.21) | <.001 | 1.91(1.65-2.22) | <.001 |
| Small-Large | 3.01(2.62-3.45) | <.001 | 2.02(1.75-2.32) | <.001 | 2.07(1.80-2.38) | <.001 |
| Teaching hospital | 2.21(2.06-2.36) | <.001 | 1.41(1.31-1.51) | <.001 | 1.42(1.32-1.53) | <.001 |
| Urban hospital | 2.31(2.00-2.66) | <.001 |  |  |  |  |
| Region of hospital |  |  |  |  |  |  |
| Northeast-Midwest | 0.94(0.84-1.04) | 0.2283 | 0.85(0.76-0.95) | 0.0039 | 0.83(0.74-0.93) | 0.0012 |
| Northeast-South | 1.36(1.24-1.49) | <.001 | 1.12(1.02-1.23) | 0.0209 | 1.08(0.99-1.19) | 0.0986 |
| Northeast-West | 0.84(0.76-0.93) | 0.0011 | 0.88(0.79-0.98) | 0.0240 | 0.88(0.78-0.98) | 0.0210 |
| Tobacco abuse | 1.45(1.32-1.59) | <.001 | 0.76(0.68-0.84) | <.001 | 0.76(0.69-0.85) | <.001 |
| Alcohol abuse | 5.21(3.74-7.25) | <.001 |  |  |  |  |
| Drug abuse | 3.96(3.53-4.44) | <.001 | 1.55(1.36-1.78) | <.001 | 1.53(1.34-1.75) | <.001 |
| Obstructive sleep apnea | 10.82(7.72-15.17) | <.001 | 0.47(0.32-0.69) | 0.001 |  |  |
| Disorders of lipoid metabolism | 7.49(5.82-9.64) | <.001 | 0.48(0.35-0.64) | <.001 |  |  |
| Nephrotic syndrome | 21.03(11.48-38.52) | <.001 |  |  |  |  |
| Chronic kidney disease | 49.22(42.29-57.29) | <.001 | 1.44(1.19-1.74) | 0.001 | 1.62(1.35-1.94) | <.001 |
| Thyrotoxicosis | 2.72(1.77-4.17) | <.001 |  |  |  |  |
| Varicose vein of lower limb | 0.00(0.00-2.995E46) | 0.8670 |  |  |  |  |
| Acquired immune deficiency syndrome | 41.31(30.80-55.41) | <.001 | 12.00(8.72-16.50) | <.001 | 11.22(8.13-15.49) | <.001 |
| Deficiency anemias | 1.87(1.72-2.04) | <.001 |  |  |  |  |
| Rheumatoid arthritis/collagen vascular diseases | 6.62(5.33-8.22) | <.001 |  |  |  |  |
| Chronic blood loss anemia | 1.93(1.79-2.08) | <.001 | 0.63(0.58-0.69) | <.001 | 0.60(0.55-0.65) | <.001 |
| Chronic pulmonary disease | 2.28(2.06-2.54) | <.001 |  |  |  |  |
| Coagulopathy | 29.91(28.07-31.87) | <.001 | 11.73(10.90-12.62) | <.001 | 11.11(10.33-11.96) | <.001 |
| Depression | 1.82(1.58-2.11) | <.001 | 0.68(0.58-0.80) | <.001 | 0.69(0.59-0.81) | <.001 |
| Hypothyroidism | 1.44(1.23-1.69) | <.001 |  |  |  |  |
| Liver disease | 12.80(10.63-15.42) | <.001 | 2.06(1.67-2.54) | <.001 | 2.15(1.75-2.64) | <.001 |
| Lymphoma | 13.85(7.35-26.10) | <.001 | 4.23(2.18-8.22) | <.001 | 4.73(2.46-9.11) | <.001 |
| Fluid and electrolyte disorders | 67.19(63.22-71.41) | <.001 | 15.31(14.18-16.53) | <.001 | 16.29(15.12-17.55) | <.001 |
| Other neurological disorders | 23.34(21.53-25.31) | <.001 | 7.45(6.78-8.20) | <.001 | 6.55(5.95-7.20) | <.001 |
| Obesity | 2.30(2.10-2.51) | <.001 |  |  |  |  |
| Paralysis | 55.83(46.53-66.98) | <.001 | 3.40(2.73-4.24) | <.001 | 2.64(2.11-3.30) | <.001 |
| Psychoses | 2.01(1.63-2.48) | <.001 | 0.62(0.49-0.78) | <.001 | 0.59(0.47-0.74) | <.001 |
| Pulmonary circulation disorders | 118.20(103.20-135.38 | <.001 | 1.39(1.17-1.67) | <.001 | 1.52(1.27-1.83) | <.001 |
| Solid tumor without metastasis | 30.10(21.50-42.15) | <.001 | 11.34(7.79-16.52) | <.001 | 11.51(7.87-16.83) | <.001 |
| Peptic ulcer disease excluding bleeding | 48.17(25.87-89.68) | <.001 | 3.87(1.92-7.79) | 0.001 | 4.52(2.27-8.99) | <.001 |
| Valvular disease | 14.47(12.41-16.87) | <.001 |  |  | 1.55(1.29-1.87) | <.001 |
| Weight loss | 58.23(50.61-67.00) | <.001 | 2.02(1.71-2.39) | <.001 | 2.36(2.01-2.78) | <.001 |
| Pulmonary embolism | 208.27(190.91-227.21 | <.001 | 20.33(18.12-22.82) | <.001 | 21.85(19.47-24.52) | <.001 |
| Congenital heart disease | 18.12(13.29-24.71) | <.001 | 1.86(1.24-2.79) | 0.0025 | 2.15(1.46-3.17) | 0.001 |
| CHA_2_DS_2_-VASc |  |  |  |  |  |  |
| Hypertension | 4.24(3.98-4.52) | <.001 | 1.73(1.61-1.86) | <.001 |  |  |
| Diabetes | 3.11(2.79-3.46) | <.001 | 1.28(1.14-1.45) | <.001 |  |  |
| Peripheral Vascular Disease | 168.08(149.56-188.89 | <.001 | 5.81(5.00-6.75) | <.001 |  |  |
| CHF | 155.77(143.52-169.06 | <.001 | 7.58(6.79-8.47) | <.001 |  |  |
| Stroke/TIA | 27.93(22.99-33.94) | <.001 |  |  |  |  |
| 1 point-2 points | 5.16(4.82-5.52) | <.001 |  |  | 2.70(2.51-2.91) | <.001 |
| 1 point-3 points | 26.31(23.95-28.90) | <.001 |  |  | 6.34(5.69-7.07) | <.001 |
| 1 point-4 points | 236.17(198.23-281.38 | <.001 |  |  | 12.50(10.11-15.46) | <.001 |
| 1 point-5 points | 458.16(306.06-685.82 | <.001 |  |  | 18.25(11.40-29.21) | <.001 |
| 1 point-6 points | 0.01(0.00-1.61E123) | 0.9748 |  |  | 0.00(0.00-2.004E55) | 0.9274 |

CHF,Chronic heart failure;TIA,Transient ischemic attack.
