# Supplementary material for: Prevalence and outcomes of atrial fibrillation in hospitalizations during pregnancy and puerperium
Source: Front Cardiovasc Med. 2026 Jul 30;13:1814450. doi: 10.3389/fcvm.2026.1814450 (PMC13469427; doi:10.3389/fcvm.2026.1814450)
Supplement: Supplementary file 8 [file Table6.docx]

**Supplement Table 6 Association of In-hospital Cardiogenic Shock with Atrial Fibrillation and Other Clinical Factors in Hospitalization During Pregnancy and Puerperium**

| Variables | Unadjusted Model | | Multivariate Model 1 | | Multivariate Model 2 | | Multivariate Model 3 | | Multivariate Model 4 | |
| --- | --- | --- | --- | --- | --- | --- | --- | --- | --- | --- |
|  | OR (95% CI) | *P* value | OR (95% CI) | *P* value | OR (95% CI) | *P* value | OR (95% CI) | *P* value | OR (95% CI) | *P* value |
| Atrial fibrillation | 24.36(22.51-26.36) | <.001 | 3.84(3.49-4.23) | <.001 | 10.85(9.97-11.80) | <.001 | 3.84(3.49-4.23) | < .001 | 3.68(3.11-4.38) | < 0.001 |
| Age group |  |  |  |  |  |  |  |  |  |  |
| 18~24-25~29 | 1.05(1.03-1.07) | <.001 | 1.05(1.03-1.07) | <.001 |  |  | 1.05(1.03-1.07) | < .001 |  |  |
| 18~24-30~34 | 1.18(1.16-1.21) | <.001 | 1.16(1.13-1.18) | <.001 |  |  | 1.16(1.14-1.19) | < .001 |  |  |
| 18~24-35~39 | 1.36(1.33-1.39) | <.001 | 1.25(1.22-1.28) | <.001 |  |  | 1.25(1.22-1.28) | < .001 |  |  |
| 18~24-40~44 | 1.76(1.70-1.83) | <.001 | 1.46(1.40-1.51) | <.001 |  |  | 1.46(1.41-1.52) | < .001 |  |  |
| 18~24-≥45 | 4.80(4.43-5.19) | <.001 | 2.29(2.09-2.50) | <.001 |  |  | 2.31(2.11-2.52) | < .001 |  |  |
| Race |  |  |  |  |  |  |  |  |  |  |
| White-Black | 1.24(1.21-1.26) | <.001 | 0.91(0.89-0.93) | <.001 |  |  | 0.94(0.92-0.96) | < .001 |  |  |
| White-Hispanic | 0.83(0.81-0.85) | <.001 | 0.91(0.89-0.93) | <.001 |  |  | 1.15(1.12-1.18) | < .001 |  |  |
| White-Other | 1.12(1.10-1.15) | <.001 | 1.12(1.09-1.15) | <.001 |  |  | 1.03(1.00-1.05) | 0.043 |  |  |
| Season |  |  |  |  |  |  |  |  |  |  |
| Winter-Spring | 1.02(1.00-1.04) | 0.1167 | 1.02(1.00-1.04) | 0.0810 |  |  | 0.98(0.96-1.00) | 0.023 |  |  |
| Winter-Summer | 0.96(0.94-0.97) | <.001 | 0.97(0.95-0.99) | 0.0021 |  |  | 1.04(1.02-1.05) | < .001 |  |  |
| Winter-Autumn | 1.05(1.03-1.07) | <.001 | 1.05(1.02-1.07) | <.001 |  |  | 0.74(0.70-0.78) | < .001 |  |  |
| Admission day is on a weekend | 1.13(1.11-1.15) | <.001 | 1.08(1.06-1.09) | <.001 |  |  | 1.07(1.05-1.09) | < .001 |  |  |
| Primary expected pay |  |  |  |  |  |  |  |  |  |  |
| Medicare-Medicaid | 0.37(0.35-0.39) | <.001 | 0.84(0.79-0.89) | <.001 |  |  | 1.07(1.05-1.08) | < .001 |  |  |
| Medicare-Private insurance | 0.38(0.36-0.39) | <.001 | 0.89(0.84-0.94) | <.001 |  |  | 1.07(1.03-1.12) | 0.0017 |  |  |
| Medicare-Self-pay | 0.40(0.37-0.42) | <.001 | 0.89(0.83-0.95) | 0.0010 |  |  | 1.18(1.13-1.23) | < .001 |  |  |
| Medicare-Other | 0.41(0.38-0.43) | <.001 | 0.98(0.91-1.05) | 0.5325 |  |  | 1.14(0.96-1.36) | 0.1325 |  |  |
| Median household income |  |  |  |  |  |  |  |  |  |  |
| <25-25~49 | 0.98(0.96-1.00) | 0.0362 | 1.04(1.02-1.06) | <.001 |  |  | 1.09(1.07-1.11) | < .001 |  |  |
| <25-50~74 | 1.03(1.01-1.05) | <.001 | 1.11(1.08-1.13) | <.001 |  |  | 1.05(1.03-1.08) | < .001 |  |  |
| <25-≥75 | 1.00(0.98-1.02) | 0.6869 | 1.08(1.05-1.10) | <.001 |  |  |  |  |  |  |
| Elective admission | 0.63(0.62-0.64) | <.001 | 0.75(0.74-0.76) | <.001 |  |  | 0.75(0.74-0.76) | < .001 |  |  |
| Control/ownership of hospital |  |  |  |  |  |  |  |  |  |  |
| Government non-federal- Private not for profit | 0.94(0.92-0.96) | <.001 | 0.92(0.90-0.94) | <.001 |  |  | 0.68(0.66-0.7) | < .001 |  |  |
| Government non-federal- Private investor owned | 0.50(0.48-0.51) | <.001 | 0.64(0.62-0.66) | <.001 |  |  | 0.62(0.55-0.69) | < .001 |  |  |
| Bed size of hospital |  |  |  |  |  |  |  |  |  |  |
| Small-Medium | 0.92(0.90-0.94) | <.001 | 0.96(0.94-0.99) | 0.0027 |  |  | 1.13(1.11-1.15) | < .001 |  |  |
| Small-Large | 1.13(1.10-1.15) | <.001 | 1.09(1.06-1.11) | <.001 |  |  |  |  |  |  |
| Teaching hospital | 1.81(1.78-1.84) | <.001 | 1.50(1.47-1.53) | <.001 | 1.67(1.64-1.70) | <.001 | 1.50(1.47-1.52) | < .001 |  |  |
| Urban hospital | 1.34(1.31-1.38) | <.001 | 0.90(0.87-0.92) | <.001 |  |  | 0.89(0.86-0.92) | < .001 |  |  |
| Region of hospital |  |  |  |  |  |  |  |  |  |  |
| Northeast-Midwest | 1.20(1.17-1.23) | <.001 | 1.21(1.19-1.24) | <.001 |  |  | 1.23(1.20-1.26) | < .001 |  |  |
| Northeast-South | 0.86(0.85-0.88) | <.001 | 0.95(0.93-0.97) | <.001 |  |  | 0.97(0.95-1.00) | 0.0194 |  |  |
| Northeast-West | 0.83(0.81-0.85) | <.001 | 0.92(0.90-0.95) | <.001 |  |  | 0.94(0.91-0.96) | < .001 |  |  |
| Tobacco abuse | 1.60(1.56-1.63) | <.001 | 1.18(1.15-1.21) | <.001 |  |  | 1.19(1.16-1.21) | < .001 |  |  |
| Alcohol abuse | 3.26(2.95-3.59) | <.001 | 1.38(1.24-1.53) | <.001 |  |  | 1.38(1.25-1.53) | < .001 |  |  |
| Drug abuse | 1.65(1.58-1.71) | <.001 | 0.93(0.89-0.97) | <.001 |  |  | 0.92(0.88-0.97) | < .001 |  |  |
| Obstructive sleep apnea | 7.38(6.70-8.13) | <.001 |  |  |  |  | 1.06(0.95-1.18) | 0.3071 |  |  |
| Disorders of lipoid metabolism | 5.03(4.68-5.40) | <.001 | 1.20(1.11-1.30) | <.001 |  |  | 1.20(1.10-1.30) | < .001 |  |  |
| Nephrotic syndrome | 5.50(4.16-7.28) | <.001 |  |  |  |  | 0.82(0.61-1.11) | 0.2014 |  |  |
| Chronic kidney disease | 11.63(10.83-12.50) | <.001 | 1.34(1.23-1.45) | <.001 |  |  | 1.35(1.24-1.47) | < .001 |  |  |
| Thyrotoxicosis | 2.00(1.78-2.25) | <.001 | 1.14(1.02-1.29) | 0.0271 |  |  | 1.14(1.01-1.29) | 0.0301 |  |  |
| Varicose vein of lower limb | 1.95(1.71-2.23) | <.001 | 1.65(1.44-1.89) | <.001 |  |  | 1.67(1.46-1.91) | < .001 |  |  |
| Acquired immune deficiency syndrome | 2.48(1.88-3.28) | <.001 |  |  |  |  | 1.10(0.82-1.46) | 0.5281 |  |  |
| Deficiency anemias | 2.72(2.67-2.77) | <.001 | 0.81(0.79-0.83) | <.001 |  |  | 0.81(0.79-0.83) | < .001 |  |  |
| Rheumatoid arthritis/collagen vascular diseases | 2.42(2.23-2.63) | <.001 |  |  |  |  | 1.03(0.94-1.12) | 0.5211 |  |  |
| Chronic blood loss anemia | 3.66(3.61-3.72) | <.001 | 3.19(3.13-3.26) | <.001 | 2.97(2.92-3.01) | <.001 | 3.18(3.12-3.25) | < .001 |  |  |
| Chronic pulmonary disease | 2.06(2.01-2.11) | <.001 | 1.32(1.29-1.36) | <.001 |  |  | 1.31(1.28-1.35) | < .001 |  |  |
| Coagulopathy | 3.71(3.60-3.82) | <.001 | 1.89(1.84-1.95) | <.001 |  |  | 1.89(1.83-1.95) | < .001 |  |  |
| Depression | 2.24(2.17-2.31) | <.001 | 1.36(1.32-1.41) | <.001 |  |  | 1.37(1.33-1.42) | < .001 |  |  |
| Hypothyroidism | 1.60(1.54-1.66) | <.001 | 1.20(1.16-1.25) | <.001 |  |  | 1.22(1.17-1.26) | < .001 |  |  |
| Liver disease | 3.05(2.79-3.33) | <.001 | 1.17(1.07-1.29) | <.001 |  |  | 1.18(1.08-1.30) | < .001 |  |  |
| Lymphoma | 2.78(1.99-3.87) | <.001 |  |  |  |  | 1.05(0.74-1.49) | 0.7707 |  |  |
| Fluid and electrolyte disorders | 15.41(15.11-15.72) | <.001 | 7.75(7.57-7.93) | <.001 | 11.70(11.46-11.94) | <.001 | 7.71(7.54-7.89) | < .001 |  |  |
| Metastatic cancer | 5.08(3.52-7.34) | <.001 |  |  |  |  | 1.12(0.77-1.64) | 0.5593 |  |  |
| Other neurological disorders | 4.31(4.15-4.48) | <.001 | 2.17(2.09-2.26) | <.001 |  |  | 2.16(2.08-2.25) | < .001 |  |  |
| Obesity | 1.71(1.67-1.75) | <.001 | 1.20(1.17-1.23) | <.001 |  |  | 1.19(1.16-1.22) | < .001 |  |  |
| Paralysis | 9.61(8.69-10.64) | <.001 | 2.28(2.04-2.56) | <.001 |  |  | 2.12(1.88-2.38) | < .001 |  |  |
| Psychoses | 2.20(2.10-2.31) | <.001 | 1.26(1.20-1.33) | <.001 |  |  | 1.28(1.22-1.35) | < .001 |  |  |
| Pulmonary circulation disorders | 13.70(12.52-14.99) | <.001 | 0.81(0.73-0.90) | <.001 |  |  | 0.82(0.73-0.91) | < .001 |  |  |
| Solid tumor without metastasis | 5.78(4.83-6.92) | <.001 | 2.80(2.33-3.38) | <.001 |  |  | 2.84(2.35-3.42) | < .001 |  |  |
| Peptic ulcer disease excluding bleeding | 8.02(5.60-11.49) | <.001 | 1.85(1.26-2.71) | 0.0016 |  |  | 1.84(1.25-2.69) | 0.0019 |  |  |
| Valvular disease | 6.05(5.73-6.39) | <.001 | 1.53(1.43-1.63) | <.001 |  |  | 1.55(1.46-1.66) | < .001 |  |  |
| Weight loss | 15.49(14.55-16.49) | <.001 | 1.98(1.85-2.12) | <.001 |  |  | 1.99(1.85-2.13) | < .001 |  |  |
| Pulmonary embolism | 15.07(14.10-16.10) | <.001 | 3.21(2.97-3.46) | <.001 |  |  | 3.19(2.96-3.44) | < .001 |  |  |
| Congenital heart disease | 5.13(4.47-5.88) | <.001 | 1.90(1.64-2.20) | <.001 |  |  | 0.71(0.51-1.00) | 0.0497 |  |  |
| CHA_2_DS_2_-VASc |  |  |  |  |  |  |  |  |  |  |
| Hypertension | 1.51(1.48-1.54) | <.001 | 0.89(0.87-0.91) | <.001 |  |  | 0.89(0.87-0.91) | < .001 |  |  |
| Diabetes | 1.72(1.66-1.78) | <.001 | 1.07(1.04-1.11) | <.001 |  |  | 1.07(1.03-1.11) | < .001 |  |  |
| Peripheral Vascular Disease | 26.29(24.59-28.11) | <.001 | 3.30(3.04-3.58) | <.001 |  |  | 3.22(2.97-3.50) | < .001 |  |  |
| CHF | 38.66(37.31-40.05) | <.001 | 6.86(6.55-7.18) | <.001 |  |  | 6.81(6.50-7.13) | < .001 |  |  |
| Stroke/TIA | 7.20(6.59-7.87) | <.001 | 1.29(1.16-1.43) | <.001 |  |  | 1.55(1.41-1.69) | < .001 |  |  |
| 1 point-2 points | 1.53(1.50-1.56) | <.001 |  |  |  |  | 3.84(3.49-4.23) | < .001 |  | |
| 1 point-3 points | 4.58(4.41-4.76) | <.001 |  |  |  |  |  |  |  | |
| 1 point-4 points | 35.81(32.93-38.95) | <.001 |  |  |  |  | 1.05(1.03-1.07) | < .001 |  | |
| 1 point-5 points | 53.32(42.39-67.08) | <.001 |  |  |  |  | 1.16(1.14-1.19) | < .001 |  | |
| 1 point-6 points | 79.78(41.23-154.38) | <.001 |  |  |  |  | 1.25(1.22-1.28) | < .001 |  | |

CHF,chronic heart failure;TIA,transient ischemic attack.
