# Supplementary material for: Prevalence and outcomes of atrial fibrillation in hospitalizations during pregnancy and puerperium
Source: Front Cardiovasc Med. 2026 Jul 30;13:1814450. doi: 10.3389/fcvm.2026.1814450 (PMC13469427; doi:10.3389/fcvm.2026.1814450)
Supplement: Supplementary file 9 [file Table7.docx]

**Supplement Table 7 Association of In-hospital Acute Respiratory Failure with Atrial Fibrillation and Other Clinical Factors in Hospitalization During Pregnancy and Puerperium**

| Variables | Unadjusted Model | | Multivariate Model 1 | | Multivariate Model 2 | | Multivariate Model 3 | | Multivariate Model 4 | |
| --- | --- | --- | --- | --- | --- | --- | --- | --- | --- | --- |
|  | OR (95% CI) | *P* value | OR (95% CI) | *P* value | OR (95% CI) | *P* value | OR (95% CI) | *P* value | OR (95% CI) | *P* value |
| Atrial fibrillation | 26.56(23.91-29.50) | <.001 | 1.16(1.01-1.33) | 0.0398 | 2.03(1.77-2.32) | <.001 | 1.08(0.94-1.25) | 0.2619 | 1.97(1.65-2.37) | <.001 |
| Age group |  |  |  |  |  |  |  |  |  |  |
| 18~24-25~29 | 0.90(0.87-0.92) | <.001 | 0.94(0.91-0.97) | <.001 |  |  | 0.94(0.91-0.96) | < .001 |  |  |
| 18~24-30~34 | 0.98(0.95-1.00) | 0.1064 | 1.03(1.00-1.06) | 0.0630 |  |  | 1.03(1.00-1.06) | 0.0946 |  |  |
| 18~24-35~39 | 1.35(1.31-1.39) | <.001 | 1.23(1.19-1.28) | <.001 |  |  | 1.23(1.18-1.27) | < .001 |  |  |
| 18~24-40~44 | 1.93(1.84-2.02) | <.001 | 1.32(1.25-1.40) | <.001 |  |  | 1.31(1.24-1.39) | < .001 |  |  |
| 18~24-≥45 | 4.09(3.65-4.60) | <.001 | 0.94(0.81-1.08) | 0.3796 |  |  | 0.90(0.78-1.04) | 0.1667 |  |  |
| Race |  |  |  |  |  |  |  |  |  |  |
| White-Black | 2.04(1.99-2.09) | <.001 | 0.85(0.82-0.87) | <.001 | 0.88(0.85-0.90) | <.001 | 0.93(0.90-0.96) | < .001 |  |  |
| White-Hispanic | 0.86(0.83-0.88) | <.001 | 0.85(0.82-0.88) | <.001 | 0.82(0.80-0.85) | <.001 | 1.27(1.22-1.31) | < .001 |  |  |
| White-Other | 1.09(1.06-1.13) | <.001 | 1.15(1.11-1.20) | <.001 | 1.14(1.10-1.18) | <.001 | 1.44(1.39-1.50) | < .001 |  |  |
| Season |  |  |  |  |  |  |  |  |  |  |
| Winter-Spring | 0.97(0.94-1.00) | 0.0255 | 0.98(0.95-1.01) | 0.2780 | 0.99(0.96-1.02) | 0.3338 | 0.94(0.91-0.96) | < .001 |  |  |
| Winter-Summer | 0.95(0.92-0.97) | 0.001 | 0.92(0.89-0.95) | <.001 | 0.93(0.90-0.96) | <.001 | 0.96(0.93-0.98) | <.001 |  |  |
| Winter-Autumn | 0.96(0.93-0.99) | 0.0079 | 0.95(0.92-0.98) | <.001 | 0.94(0.91-0.96) | <.001 | 0.88(0.82-0.94) | <.001 |  |  |
| Admission day is on a weekend | 1.31(1.28-1.34) | <.001 | 1.22(1.19-1.25) | <.001 | 1.24(1.21-1.27) | <.001 | 1.22(1.19-1.25) | < .001 |  |  |
| Primary expected pay |  |  |  |  |  |  |  |  |  |  |
| Medicare-Medicaid | 0.30(0.29-0.32) | <.001 | 1.24(1.16-1.33) | <.001 | 1.12(1.05-1.20) | <.001 | 0.84(0.81-0.86) | < .001 |  |  |
| Medicare-Private insurance | 0.18(0.17-0.19) | <.001 | 0.99(0.93-1.07) | 0.8471 | 0.93(0.86-0.99) | 0.0248 | 1.15(1.08-1.22) | < .001 |  |  |
| Medicare-Self-pay | 0.32(0.29-0.34) | <.001 | 1.43(1.31-1.56) | <.001 | 1.36(1.25-1.48) | <.001 | 1.01(0.95-1.07) | 0.795 |  |  |
| Medicare-Other | 0.25(0.23-0.27) | <.001 | 1.22(1.11-1.33) | <.001 | 1.11(1.01-1.21) | 0.0274 | 1.04(0.81-1.34) | 0.7461 |  |  |
| Median household income |  |  |  |  |  |  |  |  |  |  |
| <25-25~49 | 0.82(0.80-0.84) | <.001 | 1.02(0.99-1.05) | 0.1730 | 1.03(1.00-1.06) | 0.0675 | 0.96(0.93-0.99) | 0.003 |  |  |
| <25-50~74 | 0.72(0.70-0.73) | <.001 | 0.95(0.92-0.98) | 0.0017 | 0.97(0.94-1.00) | 0.0373 | 0.82(0.79-0.85) | < .001 |  |  |
| <25-≥75 | 0.54(0.52-0.55) | <.001 | 0.80(0.78-0.83) | <.001 | 0.83(0.80-0.86) | <.001 |  |  |  |  |
| Elective admission | 0.31(0.30-0.32) | <.001 | 0.53(0.52-0.54) | <.001 | 0.51(0.49-0.52) | <.001 | 0.53(0.52-0.55) | < .001 |  |  |
| Control/ownership of hospital |  |  |  |  |  |  |  |  |  |  |
| Government non-federal- Private not for profit | 1.01(0.98-1.04) | 0.4763 | 1.17(1.13-1.21) | <.001 | 1.19(1.15-1.23) | <.001 | 1.02(0.99-1.06) | 0.2202 |  |  |
| Government non-federal- Private investor owned | 0.89(0.85-0.92) | <.001 | 1.15(1.10-1.20) | <.001 | 1.17(1.12-1.23) | <.001 | 1.25(1.09-1.43) | 0.0012 |  |  |
| Bed size of hospital |  |  |  |  |  |  |  |  |  |  |
| Small-Medium | 1.45(1.39-1.51) | <.001 | 1.32(1.26-1.38) | <.001 | 1.33(1.27-1.39) | <.001 | 1.32(1.29-1.35) | < .001 |  |  |
| Small-Large | 2.06(1.98-2.14) | <.001 | 1.64(1.57-1.71) | <.001 | 1.65(1.59-1.72) | <.001 |  |  |  |  |
| Teaching hospital | 1.55(1.52-1.59) | <.001 | 0.96(0.93-0.98) | <.001 | 0.93(0.91-0.95) | <.001 | 0.92(0.9-0.95) | < .001 |  |  |
| Urban hospital | 2.24(2.14-2.35) | <.001 | 1.77(1.68-1.87) | <.001 | 1.77(1.68-1.86) | <.001 | 1.81(1.71-1.9) | < .001 |  |  |
| Region of hospital |  |  |  |  |  |  |  |  |  |  |
| Northeast-Midwest | 1.19(1.15-1.24) | <.001 | 0.97(0.94-1.01) | 0.1616 | 0.97(0.93-1.00) | 0.0718 | 0.91(0.88-0.95) | < .001 |  |  |
| Northeast-South | 1.38(1.33-1.42) | <.001 | 1.16(1.12-1.20) | <.001 | 1.16(1.12-1.20) | <.001 | 1.13(1.10-1.17) | < .001 |  |  |
| Northeast-West | 1.02(0.98-1.05) | 0.3473 | 0.97(0.94-1.01) | 0.1454 | 0.96(0.92-1.00) | 0.0387 | 0.94(0.91-0.98) | 0.0036 |  |  |
| Tobacco abuse | 2.66(2.60-2.73) | <.001 | 1.29(1.25-1.33) | <.001 | 1.32(1.28-1.36) | <.001 | 1.32(1.28-1.36) | < .001 |  |  |
| Alcohol abuse | 8.19(7.49-8.95) | <.001 | 1.52(1.37-1.68) | <.001 | 1.58(1.42-1.74) | <.001 | 1.51(1.36-1.67) | < .001 |  |  |
| Drug abuse | 4.71(4.55-4.88) | <.001 | 1.72(1.65-1.80) | <.001 | 1.71(1.64-1.78) | <.001 | 1.74(1.66-1.81) | < .001 |  |  |
| Obstructive sleep apnea | 34.33(32.13-36.67) | <.001 | 2.37(2.18-2.58) | <.001 | 2.78(2.56-3.02) | <.001 | 2.34(2.15-2.55) | < .001 |  |  |
| Disorders of lipoid metabolism | 7.63(7.02-8.30) | <.001 | 0.67(0.61-0.75) | <.001 | 0.80(0.73-0.89) | <.001 | 0.65(0.59-0.72) | < .001 |  |  |
| Nephrotic syndrome | 24.28(20.05-29.40) | <.001 | 1.51(1.20-1.90) | <.001 |  |  | 1.57(1.25-1.98) | 0.001 |  |  |
| Chronic kidney disease | 19.81(18.31-21.43) | <.001 | 0.52(0.48-0.58) | <.001 | 0.59(0.54-0.65) | <.001 | 0.49(0.44-0.54) | < .001 |  |  |
| Thyrotoxicosis | 4.38(3.91-4.90) | <.001 | 1.57(1.39-1.79) | <.001 | 1.62(1.43-1.84) | <.001 | 1.55(1.37-1.76) | < .001 |  |  |
| Varicose vein of lower limb | 0.70(0.51-0.96) | 0.0265 | 0.61(0.43-0.86) | 0.0045 | 0.68(0.49-0.96) | 0.0279 | 0.61(0.44-0.86) | 0.0049 |  |  |
| Acquired immune deficiency syndrome | 13.70(11.56-16.24) | <.001 | 3.65(3.01-4.44) | <.001 | 3.41(2.81-4.14) | <.001 | 3.48(2.87-4.22) | < .001 |  |  |
| Deficiency anemias | 3.65(3.56-3.73) | <.001 | 0.66(0.64-0.68) | <.001 | 0.70(0.68-0.73) | <.001 | 0.66(0.64-0.68) | < .001 |  |  |
| Rheumatoid arthritis/collagen vascular diseases | 4.68(4.29-5.10) | <.001 |  |  |  |  | 0.91(0.82-1.00) | 0.0482 |  |  |
| Chronic blood loss anemia | 5.77(5.65-5.89) | <.001 | 3.48(3.38-3.58) | <.001 | 3.32(3.22-3.41) | <.001 | 3.47(3.38-3.57) | < .001 |  |  |
| Chronic pulmonary disease | 5.13(5.00-5.26) | <.001 | 2.56(2.48-2.64) | <.001 | 2.49(2.42-2.57) | <.001 | 2.55(2.48-2.63) | < .001 |  |  |
| Coagulopathy | 14.64(14.28-15.01) | <.001 | 5.89(5.72-6.07) | <.001 | 5.59(5.43-5.75) | <.001 | 5.93(5.76-6.11) | < .001 |  |  |
| Depression | 2.77(2.66-2.88) | <.001 |  |  |  |  | 1.01(0.96-1.06) | 0.7476 |  |  |
| Hypothyroidism | 1.41(1.34-1.49) | <.001 |  |  | 0.89(0.84-0.95) | <.001 | 0.96(0.91-1.02) | 0.1773 |  |  |
| Liver disease | 6.39(5.86-6.98) | <.001 |  |  |  |  | 0.99(0.89-1.09) | 0.7926 |  |  |
| Lymphoma | 15.44(12.61-18.91) | <.001 | 4.59(3.60-5.84) | <.001 | 5.37(4.24-6.81) | <.001 | 4.69(3.69-5.97) | < .001 |  |  |
| Fluid and electrolyte disorders | 63.21(61.92-64.52) | <.001 | 16.34(15.95-16.75) | <.001 | 17.62(17.20-18.05) | <.001 | 16.26(15.86-16.66) | < .001 |  |  |
| Metastatic cancer | 25.17(19.83-31.94) | <.001 | 2.73(2.03-3.67) | <.001 | 2.73(2.04-3.65) | <.001 | 2.72(2.02-3.66) | < .001 |  |  |
| Other neurological disorders | 12.69(12.27-13.13) | <.001 | 4.09(3.93-4.26) | <.001 | 3.58(3.44-3.72) | <.001 | 4.05(3.89-4.22) | < .001 |  |  |
| Obesity | 2.94(2.86-3.02) | <.001 | 1.25(1.21-1.29) | <.001 | 1.17(1.14-1.21) | <.001 | 1.25(1.21-1.29) | < .001 |  |  |
| Paralysis | 37.16(34.46-40.08) | <.001 | 5.65(5.12-6.23) | <.001 | 4.09(3.71-4.50) | <.001 | 4.66(4.21-5.16) | < .001 |  |  |
| Psychoses | 4.28(4.08-4.50) | <.001 | 1.32(1.25-1.40) | <.001 | 1.24(1.17-1.31) | <.001 | 1.30(1.23-1.37) | < .001 |  |  |
| Pulmonary circulation disorders | 90.19(85.42-95.22) | <.001 | 1.93(1.79-2.08) | <.001 | 2.47(2.29-2.67) | <.001 | 1.88(1.74-2.03) | < .001 |  |  |
| Solid tumor without metastasis | 9.12(7.44-11.18) | <.001 | 3.35(2.65-4.22) | <.001 | 3.30(2.62-4.16) | <.001 | 3.42(2.71-4.30) | < .001 |  |  |
| Peptic ulcer disease excluding bleeding | 5.31(2.85-9.88) | <.001 | 0.24(0.11-0.51) | <.001 | 0.31(0.15-0.64) | 0.0018 | 0.25(0.12-0.52) | <.001 |  |  |
| Valvular disease | 16.35(15.57-17.17) | <.001 | 1.31(1.23-1.40) | <.001 | 2.64(2.48-2.81) | <.001 | 1.34(1.25-1.43) | < .001 |  |  |
| Weight loss | 72.87(69.71-76.18) | <.001 | 5.28(4.99-5.58) | <.001 | 5.22(4.94-5.52) | <.001 | 5.32(5.03-5.62) | < .001 |  |  |
| Pulmonary embolism | 76.72(73.33-80.28) | <.001 | 8.47(7.97-9.00) | <.001 | 8.64(8.14-9.18) | <.001 | 8.42(7.92-8.95) | < .001 |  |  |
| Congenital heart disease | 5.68(4.72-6.82) | <.001 | 0.70(0.56-0.88) | 0.0022 |  |  | 0.53(0.30-0.93) | 0.0262 |  |  |
| CHA_2_DS_2_-VASc |  |  |  |  |  |  |  |  |  |  |
| Hypertension | 5.49(5.38-5.60) | <.001 | 2.39(2.33-2.44) | <.001 |  |  | 2.37(2.32-2.43) | < .001 |  |  |
| Diabetes | 2.59(2.50-2.70) | <.001 |  |  |  |  | 1.01(0.96-1.05) | 0.7811 |  |  |
| Peripheral Vascular Disease | 59.39(55.68-63.35) | <.001 | 2.79(2.55-3.05) | <.001 |  |  | 2.52(2.31-2.76) | < .001 |  |  |
| CHF | 163.65(158.96-168.48 | <.001 | 15.99(15.35-16.66) | <.001 |  |  | 15.48(14.86-16.12) | < .001 |  |  |
| Stroke/TIA | 11.96(10.84-13.20) | <.001 | 0.66(0.58-0.75) | <.001 |  |  | 1.41(1.29-1.55) | < .001 |  |  |
| 1 point-2 points | 4.87(4.77-4.98) | <.001 |  |  | 2.77(2.71-2.84) | <.001 | 1.08(0.94-1.25) | 0.2619 |  |  |
| 1 point-3 points | 26.25(25.45-27.08) | <.001 |  |  | 8.45(8.14-8.77) | <.001 |  |  |  |  |
| 1 point-4 points | 152.73(141.72-164.60 | <.001 |  |  | 16.44(14.96-18.06) | <.001 | 0.94(0.91-0.96) | < .001 |  |  |
| 1 point-5 points | 148.46(116.50-189.18 | <.001 |  |  | 9.73(7.22-13.12) | <.001 | 1.03(1.00-1.06) | 0.0946 |  |  |
| 1 point-6 points | 0.01(0.00-2.445E39) | 0.9236 |  |  | 0.00(0.00-2.352E19) | 0.8163 | 1.23(1.18-1.27) | < .001 |  |  |

CHF,Chronic heart failure;TIA,Transient ischemic attack.
