# Supplementary material for: Prevalence and outcomes of atrial fibrillation in hospitalizations during pregnancy and puerperium
Source: Front Cardiovasc Med. 2026 Jul 30;13:1814450. doi: 10.3389/fcvm.2026.1814450 (PMC13469427; doi:10.3389/fcvm.2026.1814450)
Supplement: Supplementary file 10 [file Table8.docx]

**Supplement Table 8 Association of In-hospital Acute** **Kidney Failure with Atrial Fibrillation and Other Clinical Factors in Hospitalization During Pregnancy and Puerperium**

| Variables | Unadjusted Model | | Multivariate Model 1 | | Multivariate Model 2 | | Multivariate Model 3 | | Multivariate Model 4 | |
| --- | --- | --- | --- | --- | --- | --- | --- | --- | --- | --- |
|  | OR (95% CI) | *P* value | OR (95% CI) | *P* value | OR (95% CI) | *P* value | OR (95% CI) | *P* value | OR (95% CI) | *P* value |
| Atrial fibrillation | 48.76(44.91-52.95) | <.001 | 2.74(2.43-3.10) | <.001 | 6.28(5.62-7.00) | <.001 | 2.7(2.39-3.05) | < .001 | 1.97(1.70-2.29) | < 0.001 |
| Age group |  |  |  |  |  |  |  |  |  |  |
| 18~24-25~29 | 1.00(0.97-1.03) | 0.8544 | 0.97(0.94-1.00) | 0.0471 |  |  | 0.96(0.93-0.99) | < .001 |  |  |
| 18~24-30~34 | 1.08(1.05-1.12) | <.001 | 1.02(0.99-1.06) | 0.1938 |  |  | 1.01(0.97-1.04) | 0.735 |  |  |
| 18~24-35~39 | 1.45(1.40-1.50) | <.001 | 1.08(1.04-1.12) | <.001 |  |  | 1.07(1.03-1.11) | 0.001 |  |  |
| 18~24-40~44 | 2.30(2.19-2.41) | <.001 | 1.26(1.19-1.34) | <.001 |  |  | 1.25(1.18-1.32) | < .001 |  |  |
| 18~24-≥45 | 9.69(8.92-10.53) | <.001 | 2.08(1.86-2.32) | <.001 |  |  | 1.97(1.77-2.21) | < .001 |  |  |
| Race |  |  |  |  |  |  |  |  |  |  |
| White-Black | 3.07(2.99-3.14) | <.001 | 1.18(1.15-1.22) | <.001 | 1.45(1.41-1.49) | <.001 | 0.90(0.87-0.93) | < .001 |  |  |
| White-Hispanic | 1.11(1.07-1.14) | <.001 | 0.93(0.90-0.97) | <.001 | 0.95(0.92-0.98) | 0.0012 | 0.99(0.95-1.03) | 0.507 |  |  |
| White-Other | 1.18(1.14-1.23) | <.001 | 1.03(0.98-1.07) | 0.2280 | 1.04(1.00-1.08) | 0.0585 | 1.14(1.09-1.19) | < .001 |  |  |
| Season |  |  |  |  |  |  |  |  |  |  |
| Winter-Spring | 0.98(0.95-1.01) | 0.3014 |  |  |  |  | 1.04(1.01-1.07) | 0.0118 |  |  |
| Winter-Summer | 1.00(0.97-1.03) | 0.9776 |  |  |  |  | 1.05(1.02-1.08) | 0.009 |  |  |
| Winter-Autumn | 1.06(1.03-1.09) | <.001 |  |  |  |  | 0.90(0.83-0.97) | 0.0049 |  |  |
| Admission day is on a weekend | 1.22(1.19-1.25) | <.001 | 1.18(1.15-1.21) | <.001 |  |  | 1.18(1.15-1.21) | < .001 |  |  |
| Primary expected pay |  |  |  |  |  |  |  |  |  |  |
| Medicare-Medicaid | 0.17(0.17-0.18) | <.001 | 1.22(1.14-1.30) | <.001 |  |  | 0.91(0.88-0.93) | < .001 |  |  |
| Medicare-Private insurance | 0.11(0.10-0.11) | <.001 | 1.13(1.06-1.21) | <.001 |  |  | 1.06(1.00-1.13) | 0.0706 |  |  |
| Medicare-Self-pay | 0.18(0.17-0.19) | <.001 | 1.26(1.15-1.37) | <.001 |  |  | 0.90(0.84-0.97) | 0.0043 |  |  |
| Medicare-Other | 0.12(0.11-0.13) | <.001 | 1.11(1.01-1.22) | 0.0301 |  |  | 0.56(0.39-0.81) | 0.0019 |  |  |
| Median household income |  |  |  |  |  |  |  |  |  |  |
| <25-25~49 | 0.73(0.71-0.75) | <.001 | 0.99(0.96-1.02) | 0.4737 |  |  | 0.88(0.85-0.90) | < .001 |  |  |
| <25-50~74 | 0.63(0.61-0.65) | <.001 | 0.89(0.87-0.92) | <.001 |  |  | 0.83(0.80-0.86) | < .001 |  |  |
| <25-≥75 | 0.51(0.49-0.53) | <.001 | 0.85(0.82-0.88) | <.001 |  |  |  |  |  |  |
| Elective admission | 0.29(0.29-0.30) | <.001 | 0.53(0.52-0.55) | <.001 | 0.50(0.49-0.52) | <.001 | 0.52(0.51-0.54) | < .001 |  |  |
| Control/ownership of hospital |  |  |  |  |  |  |  |  |  |  |
| Government non-federal- Private not for profit | 0.75(0.73-0.77) | <.001 | 0.86(0.83-0.88) | <.001 |  |  | 0.96(0.92-1.00) | 0.0368 |  |  |
| Government non-federal- Private investor owned | 0.53(0.51-0.56) | <.001 | 0.84(0.81-0.89) | <.001 |  |  | 1.08(0.94-1.24) | 0.2947 |  |  |
| Bed size of hospital |  |  |  |  |  |  |  |  |  |  |
| Small-Medium | 1.39(1.33-1.45) | <.001 | 1.32(1.26-1.38) | <.001 | 1.35(1.29-1.42) | <.001 | 1.24(1.21-1.27) | < .001 |  |  |
| Small-Large | 1.96(1.89-2.04) | <.001 | 1.50(1.44-1.56) | <.001 | 1.66(1.59-1.73) | <.001 |  |  |  |  |
| Teaching hospital | 2.74(2.67-2.81) | <.001 | 1.61(1.56-1.66) | <.001 | 1.96(1.91-2.02) | <.001 | 1.62(1.57-1.67) | < .001 |  |  |
| Urban hospital | 3.30(3.12-3.50) | <.001 | 1.57(1.47-1.68) | <.001 |  |  | 1.60(1.50-1.71) | < .001 |  |  |
| Region of hospital |  |  |  |  |  |  |  |  |  |  |
| Northeast-Midwest | 1.11(1.07-1.15) | <.001 | 0.98(0.95-1.02) | 0.3635 |  |  | 0.93(0.89-0.97) | < .001 |  |  |
| Northeast-South | 1.06(1.03-1.10) | <.001 | 0.83(0.80-0.85) | <.001 |  |  | 0.85(0.82-0.88) | < .001 |  |  |
| Northeast-West | 0.88(0.85-0.92) | <.001 | 0.92(0.89-0.96) | <.001 |  |  | 0.91(0.88-0.95) | < .001 |  |  |
| Tobacco abuse | 2.01(1.95-2.07) | <.001 |  |  |  |  | 1.03(0.99-1.07) | 0.1067 |  |  |
| Alcohol abuse | 5.11(4.56-5.74) | <.001 |  |  |  |  | 0.94(0.83-1.08) | 0.3966 |  |  |
| Drug abuse | 4.10(3.94-4.26) | <.001 | 1.76(1.68-1.84) | <.001 | 1.85(1.77-1.93) | <.001 | 1.75(1.67-1.84) | < .001 |  |  |
| Obstructive sleep apnea | 25.00(23.11-27.06) | <.001 |  |  |  |  | 1.14(1.02-1.27) | 0.0168 |  |  |
| Disorders of lipoid metabolism | 16.38(15.42-17.41) | <.001 |  |  |  |  | 1.06(0.98-1.15) | 0.1592 |  |  |
| Nephrotic syndrome | 159.70(146.40-174.20 | <.001 | 4.64(4.06-5.30) | <.001 | 7.69(6.74-8.78) | <.001 | 4.70(4.12-5.37) | < .001 |  |  |
| Chronic kidney disease | 267.29(259.34-275.49 | <.001 | 19.03(18.21-19.90) | <.001 | 50.65(48.61-52.77) | <.001 | 18.87(18.05-19.72) | < .001 |  |  |
| Thyrotoxicosis | 3.32(2.90-3.79) | <.001 |  |  |  |  | 1.08(0.93-1.26) | 0.302 |  |  |
| Varicose vein of lower limb | 0.00(0.00-1.532E13) | 0.6272 |  |  |  |  |  |  |  |  |
| Acquired immune deficiency syndrome | 12.88(10.75-15.44) | <.001 | 2.33(1.86-2.91) | <.001 |  |  | 2.62(2.1-3.28) | < .001 |  |  |
| Deficiency anemias | 4.79(4.68-4.90) | <.001 |  |  |  |  | 0.90(0.87-0.94) | < .001 |  |  |
| Rheumatoid arthritis/collagen vascular diseases | 12.09(11.42-12.80) | <.001 | 1.66(1.54-1.79) | <.001 |  |  | 1.66(1.54-1.79) | < .001 |  |  |
| Chronic blood loss anemia | 5.78(5.65-5.90) | <.001 | 2.20(2.15-2.26) | <.001 | 2.29(2.23-2.34) | <.001 | 2.37(2.29-2.44) | < .001 |  |  |
| Chronic pulmonary disease | 2.43(2.34-2.51) | <.001 |  |  |  |  | 0.96(0.92-1.00) | 0.0322 |  |  |
| Coagulopathy | 15.50(15.11-15.90) | <.001 | 5.65(5.48-5.82) | <.001 | 6.34(6.16-6.53) | <.001 | 5.58(5.41-5.75) | < .001 |  |  |
| Depression | 3.18(3.05-3.31) | <.001 | 1.23(1.17-1.29) | <.001 |  |  | 1.20(1.15-1.26) | < .001 |  |  |
| Hypothyroidism | 1.92(1.83-2.02) | <.001 |  |  |  |  | 1.04(0.99-1.1) | 0.1478 |  |  |
| Liver disease | 15.65(14.76-16.61) | <.001 | 3.40(3.16-3.66) | <.001 | 3.99(3.71-4.28) | <.001 | 3.37(3.13-3.63) | < .001 |  |  |
| Lymphoma | 10.96(8.56-14.04) | <.001 | 3.74(2.82-4.97) | <.001 |  |  | 3.73(2.81-4.95) | < .001 |  |  |
| Fluid and electrolyte disorders | 90.77(88.88-92.71) | <.001 | 26.27(25.62-26.93) | <.001 | 34.90(34.06-35.76) | <.001 | 26.34(25.69-27.01) | < .001 |  |  |
| Metastatic cancer | 33.87(27.34-41.95) | <.001 | 4.40(3.41-5.69) | <.001 | 3.65(2.83-4.70) | <.001 | 4.50(3.48-5.82) | < .001 |  |  |
| Other neurological disorders | 5.75(5.47-6.04) | <.001 | 1.35(1.27-1.43) | <.001 |  |  | 1.31(1.23-1.39) | < .001 |  |  |
| Obesity | 3.21(3.12-3.30) | <.001 | 1.28(1.24-1.32) | <.001 | 2.02(1.96-2.09) | <.001 | 1.29(1.25-1.33) | < .001 |  |  |
| Paralysis | 16.31(14.54-18.29) | <.001 | 1.74(1.50-2.01) | <.001 |  |  | 1.49(1.29-1.74) | < .001 |  |  |
| Psychoses | 2.82(2.66-3.01) | <.001 |  |  |  |  | 0.96(0.89-1.03) | 0.205 |  |  |
| Pulmonary circulation disorders | 51.14(47.63-54.90) | <.001 |  |  |  |  | 1.36(1.23-1.49) | < .001 |  |  |
| Solid tumor without metastasis | 5.07(3.83-6.72) | <.001 |  |  |  |  | 1.71(1.23-2.38) | 0.0014 |  |  |
| Peptic ulcer disease excluding bleeding | 26.07(19.40-35.04) | <.001 | 3.76(2.63-5.36) | <.001 |  |  | 3.85(2.70-5.49) | < .001 |  |  |
| Valvular disease | 9.69(9.09-10.33) | <.001 |  |  |  |  | 0.90(0.83-0.98) | 0.0183 |  |  |
| Weight loss | 56.45(53.65-59.39) | <.001 | 3.12(2.93-3.33) | <.001 | 2.84(2.67-3.02) | <.001 | 3.09(2.90-3.30) | < .001 |  |  |
| Pulmonary embolism | 33.81(31.65-36.12) | <.001 | 3.31(3.05-3.59) | <.001 | 3.52(3.26-3.81) | <.001 | 3.09(2.84-3.36) | < .001 |  |  |
| Congenital heart disease | 8.01(6.82-9.41) | <.001 |  |  |  |  | 1.51(1.06-2.17) | 0.0243 |  |  |
| CHA_2_DS_2_-VASc |  |  |  |  |  |  |  |  |  |  |
| Hypertension | 9.79(9.59-10.00) | <.001 | 3.49(3.40-3.58) | <.001 |  |  | 3.53(3.44-3.62) | < .001 |  |  |
| Diabetes | 6.80(6.61-6.99) | <.001 | 1.98(1.91-2.05) | <.001 |  |  | 1.97(1.90-2.05) | < .001 |  |  |
| Peripheral Vascular Disease | 59.55(55.72-63.64) | <.001 | 2.12(1.93-2.32) | <.001 |  |  | 2.01(1.83-2.21) | < .001 |  |  |
| CHF | 115.98(112.19-119.90 | <.001 | 4.97(4.74-5.20) | <.001 |  |  | 4.92(4.69-5.16) | < .001 |  |  |
| Stroke/TIA | 21.15(19.56-22.86) | <.001 | 1.54(1.39-1.72) | <.001 |  |  | 1.83(1.66-2.01) | < .001 |  |  |
| 1 point-2 points | 8.95(8.75-9.17) | <.001 |  |  |  |  | 2.70(2.39-3.05) | < .001 |  |  |
| 1 point-3 points | 42.51(41.18-43.89) | <.001 |  |  |  |  |  |  |  |  |
| 1 point-4 points | 358.98(336.84-382.57 | <.001 |  |  |  |  | 0.96(0.93-0.99) | < .001 |  |  |
| 1 point-5 points | 742.34(634.98-867.85 | <.001 |  |  |  |  | 1.01(0.97-1.04) | 0.735 |  |  |
| 1 point-6 points | 1995.23(1293.81-3076 | <.001 |  |  |  |  | 1.07(1.03-1.11) | 0.001 |  |  |

CHF,Chronic heart failure;TIA,Transient ischemic attack.
