# Supplementary material for: Prevalence and outcomes of atrial fibrillation in hospitalizations during pregnancy and puerperium
Source: Front Cardiovasc Med. 2026 Jul 30;13:1814450. doi: 10.3389/fcvm.2026.1814450 (PMC13469427; doi:10.3389/fcvm.2026.1814450)
Supplement: Supplementary file 11 [file Table9.docx]

**Supplement Table 9 Comparison of Demographics, Hospital Characteristics, Interventions, In-Hospital Complications, and Outcomes Among Hospitalization Different CHA_2_DS_2_-VASc stratification**

| Variables | All cohort  N = 40,996,860 | CHA_2_DS_2_-VASc risk group | | | *P* value |
| --- | --- | --- | --- | --- | --- |
|  |  | Low Risk  N = 40,981,698 | Moderate Risk  N = 15,162 | High Risk  N = 15,162 |  |
| Age in years at admission | 28.32±12.95 | 28.16±12.79 | 29.42±13.69 | 34.19±14.81 | <.001 |
| Age group |  |  |  |  | <.001 |
| 18~24 | 11922245(29.08) | 10637230(29.83) | 1284249(24.10) | 767(6.79) |  |
| 25~29 | 11936750(29.12) | 10509468(29.47) | 1425346(26.75) | 1937(17.15) |  |
| 30~34 | 10693025(26.08) | 9241890(25.92) | 1447995(27.17) | 3140(27.80) |  |
| 35~39 | 5208208(12.70) | 4316515(12.11) | 888345(16.67) | 3348(29.65) |  |
| 40~44 | 1154771(2.82) | 895955(2.51) | 257362(4.83) | 1454(12.87) |  |
| ≥45 | 81861(0.20) | 55642(0.16) | 25571(0.48) | 648(5.74) |  |
| Race |  |  |  |  | <.001 |
| White | 23412509(57.11) | 20478186(57.43) | 2930381(54.99) | 3941(34.90) |  |
| Black | 5595022(13.65) | 4560802(12.79) | 1029029(19.31) | 5190(45.96) |  |
| Hispanic | 7869837(19.20) | 6963030(19.53) | 905377(16.99) | 1430(12.66) |  |
| Other | 4119493(10.05) | 3654682(10.25) | 464079(8.71) | 732(6.48) |  |
| Elderly parturient women* | 6444840(15.72) | 5268112(14.77) | 1171278(21.98) | 5449(48.25) | <.001 |
| Admission day is on a weekend | 8005528(19.53) | 7187746(20.16) | 815680(15.31) | 2102(18.61) | <.001 |
| Elective admission | 19590492(47.79) | 17134183(48.05) | 2454094(46.05) | 2214(19.61) | <.001 |
| Season |  |  |  |  | <.001 |
| Winter | 9586369(23.38) | 8316235(23.32) | 1267535(23.79) | 2599(23.01) |  |
| Spring | 9820061(23.95) | 8550755(23.98) | 1266577(23.77) | 2729(24.16) |  |
| Summer | 11571708(28.23) | 10111116(28.36) | 1457360(27.35) | 3232(28.62) |  |
| Autumn | 10018722(24.44) | 8678593(24.34) | 1337395(25.10) | 2734(24.21) |  |
| Control/ownership of hospital |  |  |  |  | <.001 |
| Government nonfederal | 5252299(12.81) | 4481055(12.57) | 769014(14.43) | 2230(19.74) |  |
| Private not for profit | 30049144(73.30) | 26163391(73.38) | 3877753(72.77) | 8000(70.84) |  |
| Private investor owned | 5695418(13.89) | 5012253(14.06) | 682101(12.80) | 1064(9.42) |  |
| Bed size of hospital |  |  |  |  | <.001 |
| Small | 5414371(13.21) | 4753100(13.33) | 660459(12.39) | 812(7.19) |  |
| Medium | 11593007(28.28) | 10125014(28.40) | 1465717(27.51) | 2276(20.15) |  |
| Large | 23989482(58.52) | 20778586(58.27) | 3202691(60.10) | 8206(72.66) |  |
| Teaching status of hospital |  |  |  |  | <.001 |
| Nonteaching | 18086372(44.12) | 16108889(45.18) | 1975288(37.07) | 2195(19.44) |  |
| Teaching | 22910488(55.88) | 19547811(54.82) | 3353580(62.93) | 9098(80.56) |  |
| Location status of hospital |  |  |  |  | <.001 |
| Rural | 4199874(10.24) | 3721345(10.44) | 478149(8.97) | 380(3.36) |  |
| Urban | 36796986(89.76) | 31935355(89.56) | 4850718(91.03) | 10913(96.64) |  |
| Region of hospital |  |  |  |  | <.001 |
| Northeast | 6659219(16.24) | 5851799(16.41) | 806033(15.13) | 1387(12.28) |  |
| Midwest | 8702310(21.23) | 7589997(21.29) | 1109688(20.82) | 2625(23.25) |  |
| South | 15716954(38.34) | 13434800(37.68) | 2276629(42.72) | 5525(48.93) |  |
| West | 9918377(24.19) | 8780104(24.62) | 1136518(21.33) | 1755(15.54) |  |
| Primary expected payer |  |  |  |  | <.001 |
| Medicare | 326638(0.80) | 252981(0.71) | 71864(1.35) | 1794(15.88) |  |
| Medicaid | 17702974(43.18) | 15407117(43.21) | 2289910(42.97) | 5946(52.65) |  |
| Private insurance | 20555598(50.14) | 17852461(50.07) | 2700148(50.67) | 2988(26.46) |  |
| Self-pay | 1180545(2.88) | 1063106(2.98) | 117108(2.20) | 330(2.92) |  |
| Other | 1231105(3.00) | 1081033(3.03) | 149837(2.81) | 235(2.08) |  |
| Median household income percentile |  |  |  |  | <.001 |
| <P25 | 11900816(29.03) | 10188381(28.57) | 1706913(32.03) | 5523(48.90) |  |
| P25~P49 | 10155987(24.77) | 8824050(24.75) | 1329081(24.94) | 2855(25.28) |  |
| P50~P74 | 9982573(24.35) | 8709511(24.43) | 1271284(23.86) | 1778(15.75) |  |
| ≥P75 | 8957484(21.85) | 7934758(22.25) | 1021589(19.17) | 1137(10.07) |  |
| Tobacco abuse | 3325478(8.11) | 2809641(7.88) | 512832(9.62) | 3004(26.60) | <.001 |
| Alcohol abuse | 67054(0.16) | 56257(0.16) | 10708(0.20) | 89(0.79) | <.001 |
| Drug abuse | 834081(2.03) | 711073(1.99) | 122159(2.29) | 849(7.52) | <.001 |
| Obstructive sleep apnea | 30988(0.08) | 12044(0.03) | 18015(0.34) | 928(8.22) | <.001 |
| Disorders of lipoid metabolism | 81150(0.20) | 41796(0.12) | 37242(0.70) | 2112(18.70) | <.001 |
| Nephrotic syndrome | 4922(0.01) | 1621(0.00) | 3146(0.06) | 155(1.38) | <.001 |
| Chronic kidney disease | 36433(0.09) | 9376(0.03) | 25112(0.47) | 1945(17.22) | <.001 |
| Thyrotoxicosis | 76189(0.19) | 61405(0.17) | 14690(0.28) | 94(0.83) | <.001 |
| Varicose vein of lower limb | 60487(0.15) | 55289(0.16) | 5189(0.10) | 10(0.09) | <.001 |
| Acquired immune deficiency syndrome | 10873(0.03) | 8746(0.02) | 2117(0.04) | 10(0.09) | <.001 |
| Deficiency anemias | 3469643(8.46) | 2921024(8.19) | 545630(10.24) | 2989(26.47) | <.001 |
| Rheumatoid arthritis/collagen vascular diseases | 125060(0.31) | 93132(0.26) | 31627(0.59) | 300(2.66) | <.001 |
| Chronic blood loss anemia | 4735410(11.55) | 3876052(10.87) | 856895(16.08) | 2463(21.81) | <.001 |
| Chronic pulmonary disease | 1714902(4.18) | 1375205(3.86) | 337388(6.33) | 2310(20.45) | <.001 |
| Coagulopathy | 733422(1.79) | 546950(1.53) | 185823(3.49) | 650(5.75) | <.001 |
| Depression | 1015765(2.48) | 815625(2.29) | 198658(3.73) | 1482(13.12) | <.001 |
| Hypothyroidism | 1111936(2.71) | 875021(2.45) | 235975(4.43) | 939(8.32) | <.001 |
| Liver disease | 89548(0.22) | 68936(0.19) | 20345(0.38) | 268(2.37) | <.001 |
| Lymphoma | 6817(0.02) | 5605(0.02) | 1197(0.02) | 15(0.13) | <.001 |
| Fluid and electrolyte disorders | 495529(1.21) | 355679(1.00) | 137218(2.57) | 2632(23.30) | <.001 |
| Metastatic cancer | 3061(0.01) | 2536(0.01) | 515(0.01) | 10(0.09) | <.001 |
| Other neurological disorders | 357436(0.87) | 270430(0.76) | 86012(1.61) | 995(8.81) | <.001 |
| Obesity | 2568669(6.27) | 1683530(4.72) | 880540(16.52) | 4599(40.72) | <.001 |
| Paralysis | 21771(0.05) | 13653(0.04) | 7687(0.14) | 431(3.81) | <.001 |
| Peripheral vascular disorders | 8407(0.02) | 0(0.00) | 7217(0.14) | 1191(10.54) | <.001 |
| Psychoses | 435042(1.06) | 352702(0.99) | 81683(1.53) | 657(5.82) | <.001 |
| Pulmonary circulation disorders | 19720(0.05) | 9098(0.03) | 10148(0.19) | 474(4.20) | <.001 |
| Solid tumor without metastasis | 11263(0.03) | 9492(0.03) | 1746(0.03) | 25(0.22) | <.001 |
| Peptic ulcer disease excluding bleeding | 2055(0.01) | 1499(0.00) | 536(0.01) | 20(0.18) | <.001 |
| Valvular disease | 119957(0.29) | 88573(0.25) | 30575(0.57) | 809(7.16) | <.001 |
| Weight loss | 36860(0.09) | 29746(0.08) | 6870(0.13) | 244(2.16) | <.001 |
| Pre-eclampsia/eclampsia | 1860449(4.54) | 0(0.00) | 1858476(34.88) | 1973(17.47) | <.001 |
| CHA_2_DS_2_-VASc |  |  |  |  | <.001 |
| Hypertension | 4413999(10.77) | 0(0.00) | 4403158(82.63) | 10842(96.00) | <.001 |
| Diabetes | 1207027(2.94) | 0(0.00) | 1198084(22.48) | 8942(79.18) | <.001 |
| Peripheral Vascular Disease | 19725(0.05) | 0(0.00) | 15754(0.30) | 3970(35.16) | <.001 |
| CHF | 52918(0.13) | 0(0.00) | 45456(0.85) | 7462(66.07) | <.001 |
| Stroke/TIA | 37475(0.09) | 56(0.00) | 34365(0.64) | 3054(27.04) | <.001 |
| CHA_2_DS_2_-VASc Score | 1.00(1.00-1.00) | 1.00(1.00-1.00) | 2.00(2.00-2.00) | 4.00(4.00-4.00) | <.001 |
| In-hospital death | 4274(0.01) | 2088(0.01) | 2027(0.04) | 160(1.42) | <.001 |
| Cardiogenic shock | 76111(0.19) | 59871(0.17) | 15568(0.29) | 671(5.95) | <.001 |
| Acute heart failure | 20152(0.05) | 0(0.00) | 17401(0.33) | 2751(24.36) | <.001 |
| Acute respiratory failure | 37754(0.09) | 18960(0.05) | 17954(0.34) | 840(7.44) | <.001 |
| Acute kidney failure | 35219(0.09) | 12626(0.04) | 21201(0.40) | 1392(12.32) | <.001 |
| Pulmonary embolism | 33639(0.08) | 25022(0.07) | 8441(0.16) | 177(1.56) | <.001 |
| Acute myocardial infarction | 3780(0.01) | 0(0.00) | 2858(0.05) | 922(8.16) | <.001 |
| Length of stay days | 2.00(2.00-3.00) | 2.00(2.00-3.00) | 3.00(2.00-4.00) | 4.00(3.00-7.00) | <.001 |
| Patient disposition |  |  |  |  | <.001 |
| Routine | 40048372(97.69) | 34888536(97.85) | 5150623(96.66) | 9213(81.58) |  |
| Transfer to Short-term Hospital | 42178(0.10) | 32259(0.09) | 9864(0.19) | 56(0.49) |  |
| Transfer to skilled nursing facility or intermediate care facility | 43780(0.11) | 34026(0.10) | 9394(0.18) | 360(3.19) |  |
| Home Health Care | 741324(1.81) | 615281(1.73) | 124924(2.34) | 1119(9.90) |  |
| Against Medical Advice | 116284(0.28) | 83937(0.24) | 31961(0.60) | 386(3.42) |  |
| In-hospital death | 4274(0.01) | 2088(0.01) | 2027(0.04) | 160(1.42) |  |
| Discharge alive destination unknown | 647(0.00) | 572(0.00) | 75(0.00) | 0(0.00) |  |
| *Elderly parturient women were defined as female during pregnancy or puerperium whose age older than 35-year-old.  Causing the weight of observation may not be integer, the count of each group may less or more than the total of all group after rounding off. CHF,Chronic heart failure;TIA,Transient ischemic attack. | | | | | |
